# Identification and Characterization of circRNAs in Non-Lactating Dairy Goat Mammary Glands Reveal Their Regulatory Role in Mammary Cell Involution and Remodeling

**DOI:** 10.3390/biom13050860

**Published:** 2023-05-18

**Authors:** Rong Xuan, Jianmin Wang, Qing Li, Yanyan Wang, Shanfeng Du, Qingling Duan, Yanfei Guo, Peipei He, Zhibin Ji, Tianle Chao

**Affiliations:** Shandong Provincial Key Laboratory of Animal Biotechnology and Disease Control and Prevention, Shandong Agricultural University, 61 Daizong Street, Tai’an 271018, China; 2018110375@sdau.edu.cn (R.X.); wangjm@sdau.edu.cn (J.W.); 2022010086@sdau.edu.cn (Q.L.); zbji916@sdau.edu.cn (Z.J.)

**Keywords:** circRNAs, dairy goat, non-lactation, mammary gland involution, cell remodeling

## Abstract

This study conducted transcriptome sequencing of goat-mammary-gland tissue at the late lactation (LL), dry period (DP), and late gestation (LG) stages to reveal the expression characteristics and molecular functions of circRNAs during mammary involution. A total of 11,756 circRNAs were identified in this study, of which 2528 circRNAs were expressed in all three stages. The number of exonic circRNAs was the largest, and the least identified circRNAs were antisense circRNAs. circRNA source gene analysis found that 9282 circRNAs were derived from 3889 genes, and 127 circRNAs’ source genes were unknown. Gene Ontology (GO) terms, such as histone modification, regulation of GTPase activity, and establishment or maintenance of cell polarity, were significantly enriched (FDR < 0.05), which indicates the functional diversity of circRNAs’ source genes. A total of 218 differentially expressed circRNAs were identified during the non-lactation period. The number of specifically expressed circRNAs was the highest in the DP and the lowest in LL stages. These indicated temporal specificity of circRNA expression in mammary gland tissues at different developmental stages. In addition, this study also constructed circRNA–miRNA–mRNA competitive endogenous RNA (ceRNA) regulatory networks related to mammary development, immunity, substance metabolism, and apoptosis. These findings help understand the regulatory role of circRNAs in mammary cell involution and remodeling.

## 1. Introduction

The mammary gland is a complex exocrine gland composed of many different types of cells that distinguish mammals from other animals by its unique anatomy [1]. It can synthesize, secret, and deliver milk to newborns for optimal protection, nutrition, and development [2]. Affected by the gestational cycle, the adult mammary gland undergoes periodic developmental changes of pregnancy–lactation–involution in the life of female mammals [3]. The active involution of the mammary gland occurs during the non-lactating period and is a highly complex multi-step process [4]. It occurs when the lactating mammary gland returns to a morphologically close pre-pregnancy state after forced or natural weaning [5]. Active involution is characterized by a high degree of epithelial cell apoptosis [6], increased permeability of interepithelial junctions [7], activation or inactivation of various proteases and their inhibitors [8], increased numbers of neutrophils and macrophages [9], changes in milk composition [10], and remodeling of mammary adipose tissue [11]. In the process of mammary gland involution to lactation again, mammary cells undergo apoptosis and renewal to realize the self-renewal and repair of the mammary gland tissue, namely mammary gland remodeling [12]. Thus, the involution of non-lactating mammary tissue and the remodeling of mammary cells is critical for maintaining mammary health, milk production, and fertility in the subsequent lactation.

Mammary gland involution is regulated by multiple factors, including some growth factors, proteases, hormones, coding genes, and non-coding RNAs (ncRNAs) [3,5,13]. circRNA is a type of ncRNAs that is a covalently closed circular transcript generated by back splicing [14]. It has the characteristics of a stable structure, rich variety, and evolutionary conservation [15,16,17]. The expression of circRNAs has time-and tissue-specific characteristics. For example, circRNAs are differentially expressed in mammary gland tissues at different developmental stages, which has been confirmed in mammalian mammary gland tissues, such as that from goats [18], sheep [19,20], cattle [21,22], and mice [23], by transcriptome sequencing. In addition, the number of circRNAs in human glandular tissues (adrenal, mammary gland, pancreas, and thyroid), especially in the mammary gland (9665 candidate circRNAs), was higher than that in other adult tissues (colon, heart, kidney, liver, lung, and stomach) [24]. circRNA also has multiple mechanisms of action, such as regulating gene transcription, acting as a molecular sponge of miRNA and transcriptional regulator, interacting with RNA-binding protein (RBP), and participating in protein translation and immunomodulation [25,26]. Current studies have shown that circRNA can act as a proliferation regulator of mammary epithelial cells and participate in the synthesis of milk protein and milk fat [21,27,28]. Furthermore, cadmium promotes apoptosis and inflammation in mammary epithelial cells of bovine and mice through the circ08409/miR-133a/TGFB2 axis [29]. This indicates that circRNAs play an important role in the regulation of mammary gland development and lactation.

However, the expression characteristics and molecular functions of circRNAs in goat-mammary-gland involution are still unclear, although current studies have characterized circRNAs in both lactating and non-lactating mammary gland tissues of sheep [20] and cattle [22]. Mammary gland involution is a continuous and complex multi-step process [4], and setting more sampling and sequencing time points in non-lactation may help to better explain the molecular mechanism behind the physiological changes in mammary gland involution and remodeling process [30]. Furthermore, the degree and rapidity of mammary gland involution vary between species [3]. There is currently insufficient information about the function of circRNAs in goat-mammary-gland involution. Therefore, this study performed non-lactating (LL, DP, and LG) goat-mammary-gland transcriptome sequencing to reveal the expression characteristics of circRNAs in non-lactating goat-mammary-gland tissue and to screen circRNAs related to mammary gland development and lactation regulation. This provides new insights into the regulatory roles of circRNAs in mammary cell involution and remodeling.

## 2. Materials and Methods

### 2.1. Ethics Statement

The methods and experimental procedures used in this study were approved and directed by the animal protection and ethics committee of Shandong agricultural university (protocol number: SDAUA-2018-048) [13]. All experimenters underwent strict training and self-protection to minimize the pain experienced by animals.

### 2.2. Experimental Animal Management and Sample Collection

Nine healthy Laoshan dairy goats raised at the Qingdao Aote goat farm (Qingdao, China) were used in this study. Similar standards of housing and management were provided for all goats. An open-air natural sports field was provided to keep the breeding environment clean, hygienic, and dry. The goats were fed according to the standard feeding process of the farm. The goats were all in the third parity, with an age range of 3 years and 8 months to 4 years and 1 month; the weight range of the goats was 50.16 to 56.41 kg; and the specific information of the goats can be obtained in Table S1 of our previous study [31]. Mammary gland tissue from the ipsilateral breast was collected during late lactation (LL, *n* = 3, 240 days postpartum), dry period (DP, *n* = 3, 300 days postpartum), and late gestation (LG, *n* = 3, 140 days after mating). The specific tissue collection method was conducted as follows: after intravenous injection of sodium pentobarbital (100 mg/kg), the muscles relaxed, and the heart and respiration stopped; the goats were then rapidly dissected; and mammary gland tissue was harvested. Immediately after washing the mammary tissue with normal saline (0.9%), the harvested tissue was placed into an RNase-free cryovial and stored in liquid nitrogen.

### 2.3. RNA Extraction and Sequencing Analysis

Total RNA was extracted using TRIzol-based RNAiso Plus (Code: 9108Q, Takara, Beijing, China). The quality of RNA was analyzed using a NanoDrop 2000C (Thermo Fisher Scientific, Wilmington, NC, USA). Only when the RNA integrity number (RIN) was > 8 were the RNA samples used for subsequent experimental analysis. Nine sequencing libraries (LL1, LL2, LL3, DP1, DP2, DP3, LG1, LG2, and LG3) were constructed using the TruSeq RNA Library Prep Kit v2 (Illumina, San Diego, CA, USA), and they were sequenced on the HiSeq 2500 platform (Illumina). After generating raw data, the sequencing data quality was assessed using FastQC software (version 0.11.9; https://www.bioinformatics.babraham.ac.uk/projects/fastqc/, accessed on 15 January 2023) and high-throughput sequencing reads were processed using Trimmomatic software (version 0.39) [32] to remove adapter sequences, primers, poly-A tails, and low-quality sequences. Using find_circ [33], CIRIquant [34], CIRCexplorer [35], and CIRI2 [36] to identify circRNAs, the intersection of the circRNAs identified by the four software was taken for subsequent quantitative analysis, and CIRIquant was used to quantify circRNAs. The main steps are as follows: HISAT2 [37] or BWA [38] software was used to construct the goat genome index and sequence alignment. The NCBI BioSample number for the goat reference genome was SAMN03863711. SAMtools was used to convert the aligned sam files into bam format files [39]. StringTie was used to assemble transcripts and calculate the expression of circRNAs [40].

### 2.4. Classification of circRNAs and Functional Analysis of Their Source Genes

Low-expression circRNAs were filtered and transcripts with expression levels ≥ 2 were retained. Principal component analysis (PCA) was performed on all circRNAs and the expressed circRNAs in each mammary-gland-developmental stage were counted. circRNAs were classified according to their position information in the genome, and circRNAs types are displayed in a Venn plot. In addition, the length distribution of circRNA is displayed by bar graph. Gene ontology (GO) functional annotation and Kyoto encyclopedia of genes and genomes (KEGG)-pathway-enrichment analysis were performed on circRNA-derived genes using clusterProfiler software [41]. Goat genes annotated in the GO and KEGG databases were used as background genes for enrichment analysis. GO terms and KEGG pathways were considered significantly enriched when they met false discovery rate (FDR) < 0.05. According to the previously identified up-regulated and down-regulated differentially expressed genes in the goat mammary gland [13], the GO bubble plot and GO circular plot were drawn using GOplot software [42] to show the significantly enriched GO terms in three comparisons (LL vs. DP, DP vs. LG, and LL vs. LG).

### 2.5. Differential Expression Analysis of circRNAs and Real-Time Quantitative Reverse-Transcription PCR Assay

Differential expression analysis (LL vs. DP, DP vs. LG, and LL vs. LG) of mammary gland tissues at different stages was performed using edgeR software [43]. When the adjusted *p* value (*p*.adj) < 0.05 and the absolute value of log2 (fold change) ≥ 1, circRNAs were considered differentially expressed. According to the ranking of *p*.adj, the 10 most significantly up-regulated and down-regulated circRNAs were screened and are displayed in a volcano map. The differentially expressed circRNAs were then sorted according to the expression level at each developmental stage, and the top 10 differentially expressed circRNAs at each developmental stage (LL, DP, and LG) were selected and are displayed using histograms. The R package pheatmap was used to draw a heatmap to display the expression characteristics of all differentially expressed circRNAs.

To verify the accuracy and reliability of transcriptome data, we randomly selected 14 differentially expressed circRNAs for real-time quantitative reverse-transcription PCR (RTqPCR) assay and loop junction site verification. Total RNA was extracted from goat-mammary-gland tissue using TRIzol-based RNAiso Plus (Code: 9108Q, Takara). Linear RNA was removed using Ribonuclease R (Cat.No.R0301, Geneseed, Guangzhou, China). Reverse-transcription reactions were performed using the RevertAid First Strand cDNA Synthesis Kit (K1622, Invitrogen, Vilnius, Lithuania) according to the manufacturer’s instructions. PCR reactions were performed using the 2xTaq MasterMix kit (Vazyme, Nanjing, China). PCR products were confirmed by 2% agarose gel electrophoresis. Bands approximately 100 bp in length were isolated and analyzed by Sanger sequencing. In order to determine the expression level of circRNAs, TB Green^®^ Fast qPCR Mix (Takara) was used for the real-time quantitative reverse-transcription PCR (RTqPCR) assay. Primers were designed using NCBI Primer Blast software, and the specific steps are as follows: after obtaining the circRNA sequences, the sequence (100 bp) at the 3′ end was placed in front of the 100 bp at the 5′ end of the sequence. The new sequence (200 bp) was uploaded to the NCBI Primer Blast website, the length of the PCR product was set to 90–120 bp, and the divergent primer of the circRNA was obtained. The geometric mean of 2 carefully selected reference genes (*GAPDH* and *MRPL39*) was used as an accurate normalization factor [13]. Relative gene expression was calculated using the improved Pfaffl Method [44].

### 2.6. Expression Patterns of Differentially Expressed circRNAs and Functional Analysis of Genes in ceRNA Network

miRanda software [45] was used to predict the targeting relationship between miRNAs and differentially expressed circRNAs or mRNAs. The energy threshold is set to −10 and the score threshold is set to 150. Expression cluster analysis of differentially expressed circRNAs was performed using R package TCseq. clusterProfier software was used to perform GO functional annotation and KEGG-pathway-enrichment analysis on genes with the circRNA–miRNA–mRNA (ceRNA) relationship in each cluster. Bar plots and dot plots are used to display the GO and KEGG analysis results, respectively.

### 2.7. Construction of circRNA–miRNA–mRNA ceRNA Regulatory Network

The function corr.test in the R package psych was used to calculate the Pearson correlation between miRNA and circRNA, miRNA and mRNA, and circRNA and mRNA. The correction method used was FDR. Relationship pairs with a correlation value ≥ 0.8 and an FDR < 0.05 between circRNA and mRNA, a correlation ≤ −0.8 and an FDR < 0.05 between miRNA and circRNA, and a correlation value ≤ −0.8 and an FDR < 0.05 between miRNA and mRNA were screened. Considering the target genes results predicted by miRanda software and the expression correlation between miRNAs and circRNAs or mRNAs comprehensively, GO functional annotation and KEGG-pathway-enrichment analysis were performed on mRNAs that compete with differentially expressed circRNAs for miRNA binding. The signaling pathways and genes related to physiological processes, such as mammary gland development, cell apoptosis, immunity, and substance metabolism, were screened out. STRING software [46] was used to construct protein–protein interaction (PPI) networks. Cytoscape software [47] was used to present the PPI network and calculate each note degree through the built-in function network analyzer. According to the note degree, the hub gene in each functional network was determined. The circRNA–miRNA–mRNA ceRNA regulatory network was constructed by Cytoscape software. Histograms and heatmaps were used to display the expression levels of hub genes and their associated miRNAs and circRNAs in the ceRNA network at three developmental stages.

### 2.8. Statistical Analysis

Student’s *t*-test was used to test the significance of the difference between the experimental group and the control group; *p* < 0.05 indicated a significant difference. Tukey’s honestly significant-difference test was used to analyze gene expression levels at different developmental stages. Figures presented in this work were generated using R software (version 4.2.0) unless otherwise stated.

## 3. Results

### 3.1. Identification and Classification of circRNAs

**Figure 1:**
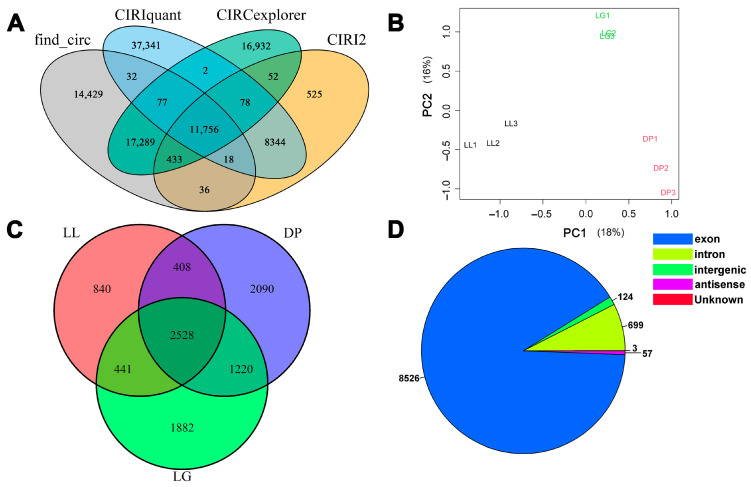
Identification and classification of circRNAs. (**A**) Number of circRNAs identified by four software; (**B**) Principal component analysis of mammary gland tissue samples; (**C**) Venn diagram of circRNA expression at different developmental stages; (**D**) Classification of circRNAs. LL: late lactation; DP: dry period; and LG: late gestation.

Statistical analysis of the sequencing data found that 75,670,537 ± 2,451,052, 80,547,045 ± 4,443,268, and 73,765,766 ± 1,723,917 clean reads (mean ± SE) were obtained from the sequencing library of goat mammary glands during late lactation (LL), dry period (DP), and late pregnancy (LG), respectively. The results of the reads’ alignment with the goat reference genome showed that the alignment rate of each sequencing library was ≥ 95% (Table S1). Raw sequencing data were submitted to the NCBI GEO database (GEO accession number: GSE185981). This indicates that library construction and the sequencing of the non-lactating goat mammary gland was successful. According to the intersection results of the four software, a total of 11,756 circRNAs were identified, of which 9409 circRNAs had an expression level of FPKM ≥ 2 (Figure 1A and Table S2). The results of the PCA showed that mammary gland tissue samples in the three periods could be identified according to principal component 1 and principal component 2. Mammary tissue samples from the same period were clustered together (Figure 1B). According to the analysis of the expression levels of circRNAs in each period, it was found that 2528 circRNAs were expressed in the three periods (LL, DP, and LG). The number of circRNAs exclusively expressed in the DP stage was the highest, and the number of circRNAs exclusively expressed in the LL stage was the lowest (Figure 1C). These indicated temporal specificity of circRNA expression in mammary gland tissues at different developmental stages. According to the location (Figure 1D) in the genome, these circRNAs can be classified into exonic, intergenic, antisense, and intronic circRNAs. Among them, the number of circRNAs derived from exons is the largest (accounting for 90.6% of all circRNAs), and the least identified circRNAs are antisense circRNAs. From circRNA length distribution (Figure S2), the length of circRNAs ranged from 141 to 187,697 nucleotides (nt), of which 50.52% were larger than 10,000 nt. For circRNAs smaller than 10,000 nt, circRNAs with a length of 200–4000 nt accounted for 26.17% of all circRNAs. The number of circRNAs identified from chromosomes 1 and 3 was the highest (Figure S1A). In addition, the chromosomal origin information of 271 circRNAs was unknown (Table S2).

### 3.2. Functional Analysis of circRNAs’ Source Genes

**Figure 2:**
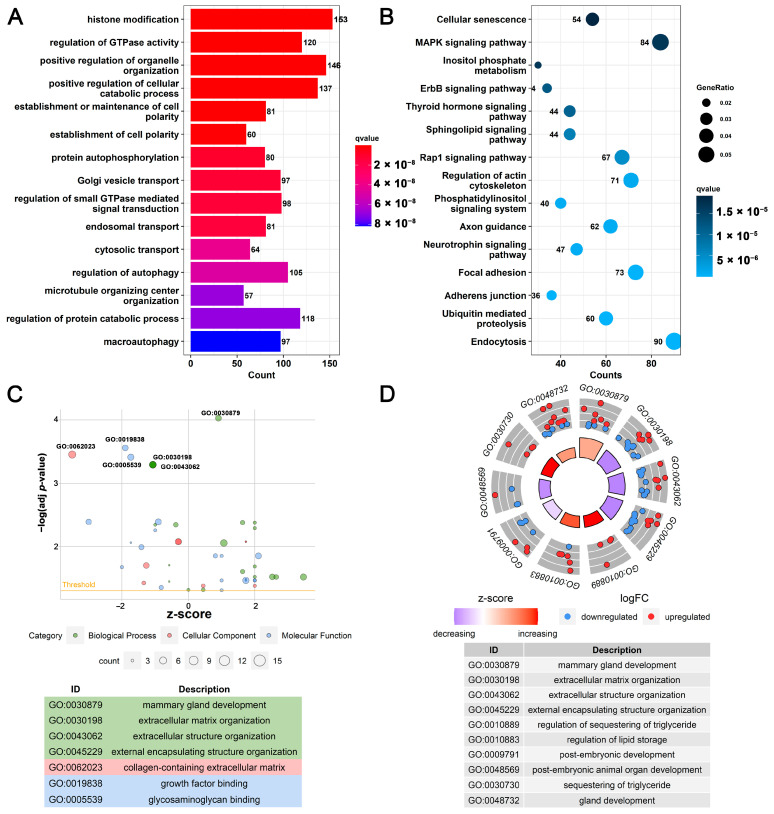
GO annotation and KEGG-pathway-enrichment analysis of source genes of circRNAs. (**A**) GO annotation analysis of circRNAs’ source genes; (**B**) KEGG-pathway-enrichment analysis of circRNAs’ source genes; (**C**) A bubble plot of GO functions’ annotation analysis of up-regulated and down-regulated circRNAs’ source genes in LL vs. DP; (**D**) A circular plot of GO functions annotation analysis of up-regulated and down-regulated circRNAs’ source genes in LL vs. DP. LL: late lactation; DP: dry period; and LG: late gestation.

Analysis of 9409 circRNAs revealed that 9282 circRNAs were derived from 3889 genes, and 127 circRNAs were of unknown origin. There were 1844 (47.4%) genes that produced 1 circRNA transcript, and only 45 (1.2%) genes could produce 10 or more circRNA transcripts (Figure S1B). The genes (*LALBA*, *CSN1S1*, *CSN1S2*, *CSN2*, and *CSN3*) affecting protein content and composition in milk could produce circRNAs, of which 10 circRNAs were derived from *CSN1S2* and 8 circRNAs were derived from *CSN2*. GO functional analysis of the 3889 cicrRNA-derived genes found (Figure 2A), histone modification, regulation of GTPase activity, positive regulation of organelle organization, establishment or maintenance of cell polarity, and other GO terms were significantly enriched (FDR < 0.05). KEGG analysis results showed that (Figure 2B) endocytosis, ubiquitin-mediated proteolysis, adherens junction, the ErbB signaling pathway, the MAPK signaling pathway, cellular senescence, and other signaling pathways were significantly enriched (FDR < 0.05). This reflects the functional diversity of circRNAs’ source genes. In addition, GO functional analysis of differentially expressed circRNA-derived genes found (Figure 2C,D and Table S3) in the LL vs. DP group, mammary gland development, extracellular matrix organization, regulation of lipid storage, post-embryonic development, and other GO terms related to cell growth and development were significantly enriched (FDR < 0.05). Among them, the number of up-regulated genes in pathways, such as mammary gland development, gland development, sequestering of triglyceride, regulation of lipid storage, and regulation of sequestering of triglyceride, is more than the number of down-regulated genes. In the DP vs. LG group (Figure S3A), mammary gland development, mammary gland epithelium development, wound healing, lactation, and other GO terms were significantly enriched (FDR < 0.05). Interestingly, genes (*CSN2*, *CSN3*, *ERBB4*, *PRLR*, and *XDH*) in the lactation term were down-regulated during the DP. This may be related to the lack of milk production during the DP. The number of genes up-regulated during the DP in wound healing was more than the number of down-regulated genes, which may be related to tissue remodeling during the DP. In the LL vs. LG group (Figure S3B), mammary gland development, response to peptide hormone, response to insulin, and other GO terms were significantly enriched (FDR < 0.05). In addition, the results of GO functional analysis on the up-regulated/down-regulated genes of differentially expressed circRNAs are provided in Figures S4–S6.

### 3.3. Differential Expression Analysis Results and Verification of circRNA Expression by RTqPCR

**Figure 3:**
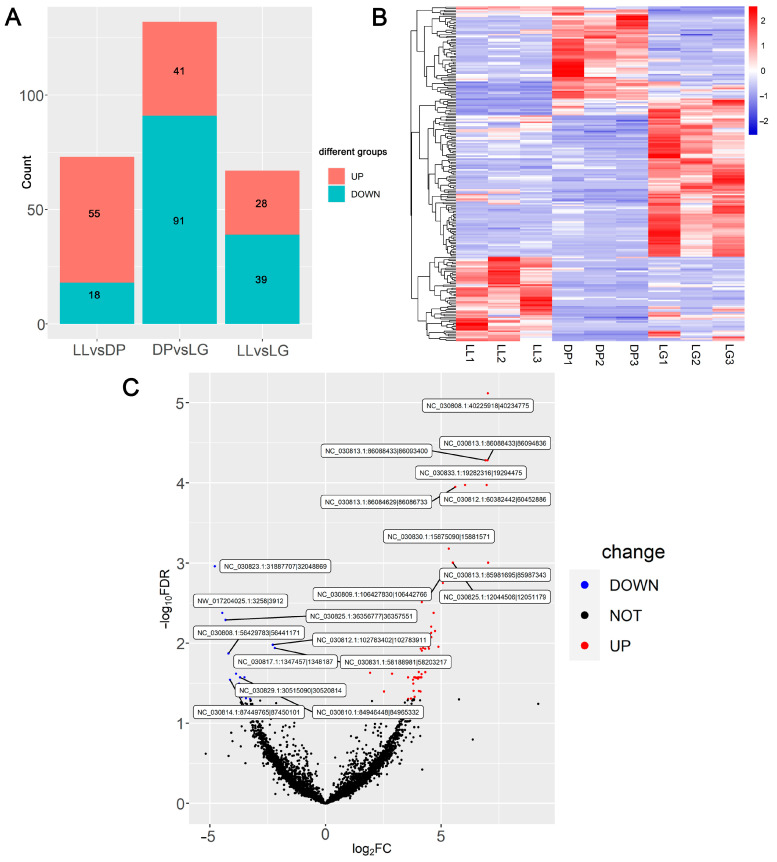
Differential expression analysis of circRNAs in different mammary-gland-developmental stages. (**A**) Number of up-regulated and down-regulated circRNAs; (**B**) A heatmap of differentially expressed circRNA expression; (**C**) Volcano map of differentially expressed circRNAs in LL vs. DP. LL: late lactation; DP: dry period; and LG: late gestation.

The results of differential expression analysis showed that a total of 218 differentially expressed circRNAs were identified in the three comparison groups (Table S4). Among them, the number of differentially expressed circRNAs was the largest in the DP vs. LG comparison (Figure 3A). A heatmap (Figure 3B) was used to show the expression changes of 218 circRNAs at three mammary-gland-developmental stages. According to the volcano plot of differentially expressed circRNAs (Figure 3C), 55 circRNAs were up-regulated and 18 circRNAs were down-regulated in the comparison between LL and the DP. Among them, the differential expression folds of 11 circRNAs were higher than 30, and the change in the expression level of *NC_030813.1:85981695|85987343* was the largest at 130.2-fold. In the DP vs. LG comparison (Figure S7A), 41 circRNAs were up-regulated and 91 circRNAs were down-regulated. The down-regulated circRNA *NC_030833.1:19282316|19294475* showed the largest change at 58.5-fold. In the comparison of LL vs. LG (Figure S7B), 28 circRNAs were up-regulated, 39 circRNAs were down-regulated, and *NC_030813.1:86088433|86094836* was up-regulated by 63.2-fold. In addition, the expression histogram shows the top 10 highly expressed circRNAs in each period (Figure S8). In summary, the above indicated that the transcriptome-level expression of circRNAs changed during the involution and remodeling of mammary gland tissue, indicating the stage specificity of circRNA expression in mammary gland tissue.

In addition, the results of RNA-Seq (log2 fold change) and RTqPCR (log2 fold change) of 14 circRNAs were significantly positively correlated (correlation value = 0.79, *p*-value < 0.01) (Figure S9). Agarose gel electrophoresis of 2% showed that the length of the single band of each selected circRNA was in line with the length of the expected design amplification (Figure S10B). The results of Sanger sequencing (Figures S10C and S11) found that the circular junction sequence information obtained by Sanger sequencing was completely consistent with the results of circRNA transcriptome sequencing. This indicates that the results of transcriptome sequencing and differential expression analysis in this study are reliable. All primers of circRNAs for RTqPCR are provided in Table S5.

### 3.4. Expression Patterns of Differentially Expressed circRNAs and circRNA–miRNA–mRNA ceRNA Relationship Analysis

**Figure 4:**
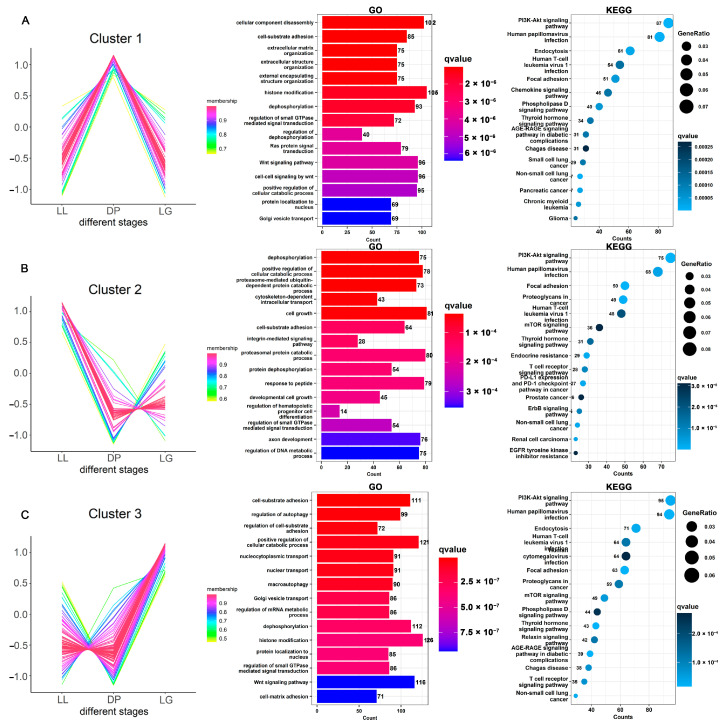
Expression patterns and target-gene-function analysis of differentially expressed circRNAs. Cluster analysis was performed on 218 circRNAs according to their expression levels, and finally, 3 cluster lncRNAs were obtained. (**A**–**C**) shows cluster1, cluster2, and cluster3 of circRNAs, respectively. According to the circRNA–miRNA–mRNA ceRNA relationship and co-expression relationship, the target genes corresponding to the circRNAs in each cluster were obtained. The bar graphs and bubble plots show the GO annotation and KEGG-pathway-enrichment analysis results of target genes corresponding to circRNAs, respectively. LL: late lactation; DP: dry period; and LG: late gestation.

A total of 23,574 target regulatory relationships were predicted between 330 miRNAs and 218 differentially expressed circRNAs by miRanda software. There were 441,782 targeted regulatory relationships between miRNAs and 12,056 protein-coding transcripts (Table S6). Through cluster analysis of circRNA expression, circRNAs were clustered into three clusters (Figure 4 and Table S7). The first cluster contained 58 circRNAs, which were highly expressed in the DP. The corresponding functional analysis results of miRNA target genes (Figure 4A) showed that GO terms, such as cellular component disassembly, cell–substrate adhesion, and extracellular matrix organization, were significantly enriched (FDR < 0.05). The KEGG pathway analysis results showed that the PI3K-Akt signaling pathway, thyroid hormone signaling pathway, and focal adhesion were significantly enriched (FDR < 0.05). In addition, signaling pathways related to immunity and diseases (human papillomavirus infection, non-small-cell lung cancer, chemokine signaling pathway, etc.) were also significantly enriched during the DP. The second cluster contained 54 circRNAs that were highly expressed during LL (Figure 4B). GO results showed that dephosphorylation, positive regulation of cellular catalytic process, cell growth, extrinsic apoptotic signaling pathway, and other GO terms are significantly enriched (FDR < 0.05). KEGG pathway results showed that the PI3K-Akt signaling pathway, ErbB signaling pathway, mTOR signaling pathway, apoptosis, and other pathways related to cell growth were significantly enriched. The largest number of circRNAs in cluster 3 was 106 circRNAs, which were highly expressed in LG (Figure 4C). Cell–substrate adhesion, regulation of autophagy, nucleocytoplasmic transport, Golgi vesicle transport, dephosphorylation, histone modification, the prolactin signaling pathway, the insulin signaling pathway, and other terms were significantly enriched (FDR < 0.05). In summary, circRNAs can affect physiological processes, such as apoptosis, growth, substance metabolism, tissue remodeling, intercellular adhesion, intercellular connection and communication, disease infection, and immune response, by competitively binding with miRNAs.

### 3.5. Analysis of circRNA–miRNA–mRNA ceRNA Regulatory Network Related to Mammary Gland Development

**Figure 5:**
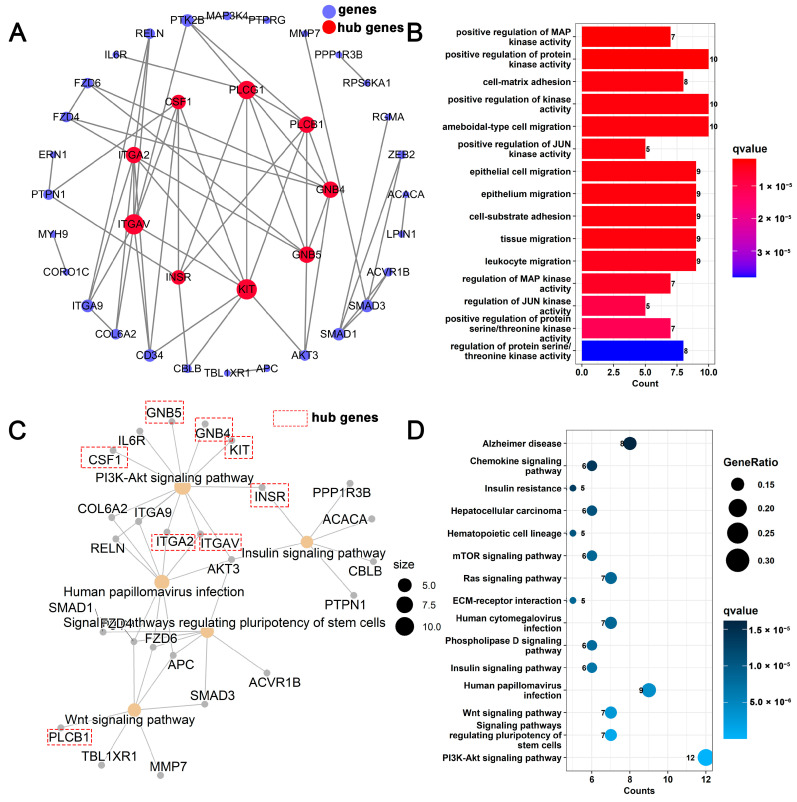
Analysis of circRNA–miRNA–mRNA ceRNA regulatory network related to mammary gland development. (**A**) PPI network analysis of mammary-gland-development-related genes; (**B**) GO analysis of mammary-gland-development-related genes; (**C**) Relationship between KEGG pathways and genes; (**D**) KEGG analysis of mammary-gland-development-related genes.

**Figure 6:**
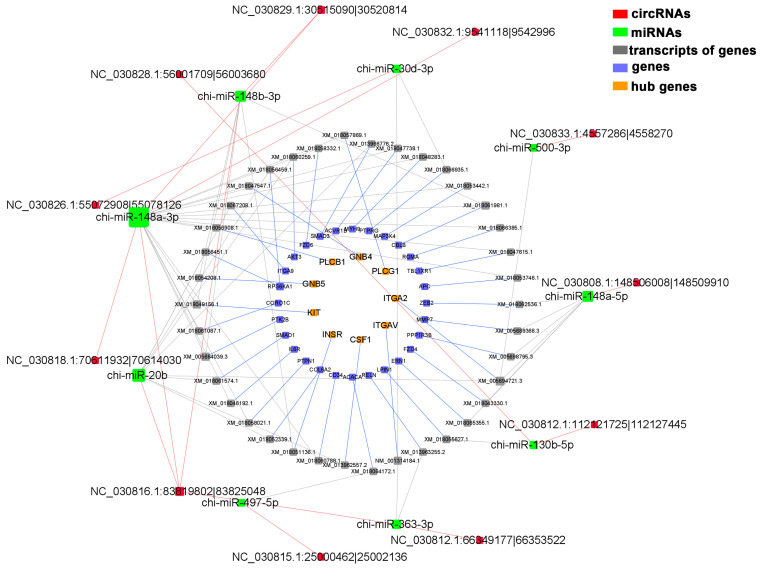
Analysis of circRNA–miRNA–mRNA ceRNA regulatory network related to mammary gland development.

**Figure 7:**
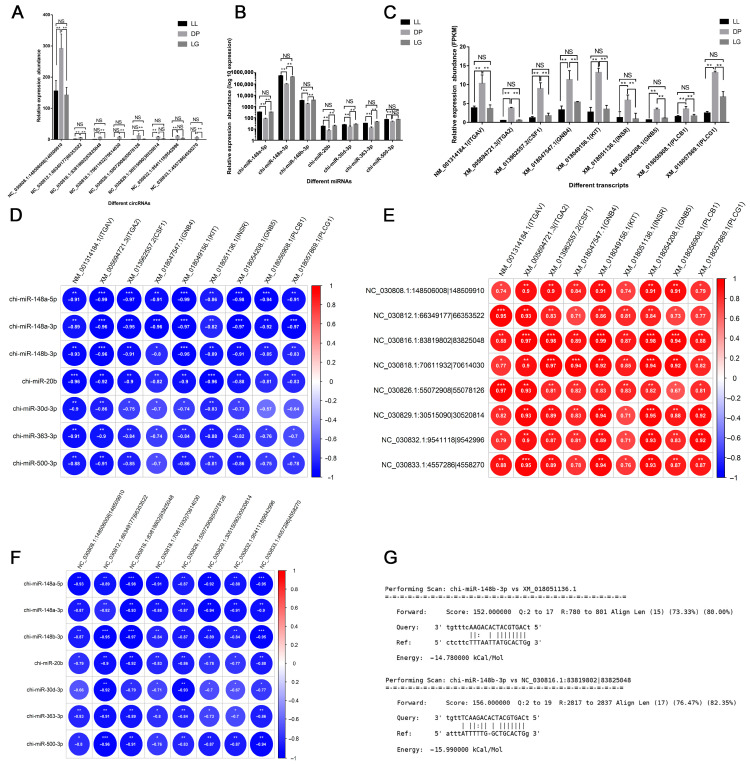
Analysis of expression patterns of circRNAs related to mammary gland development. (**A**) Analysis of circRNAs’ expression pattern related to mammary gland development; (**B**) Analysis of miRNAs’ expression pattern related to mammary gland development; (**C**) Analysis of mRNAs’ expression pattern related to mammary gland development; (**D**) Correlation between miRNAs’ and mRNAs’ expression; (**E**) Correlation between circRNAs’ and mRNAs’ expression level; (**F**) Correlation between miRNAs’ and circRNAs’ expression level; (**G**) Target relationship predicted by miRanda software. * indicates *p* < 0.05, ** indicates *p* < 0.01, *** indicates *p* < 0.001, and NS indicates not significant.

A total of 37 genes related to mammary gland development were screened, and 9 genes were identified as hub genes of the PPI network (Figure 5A). The results of GO analysis showed (Figure 5B) that these genes were involved in physiological processes, such as positive regulation of MAP kinase activity, cell–matrix adhesion, and epithelial cell migration. KEGG analysis showed (Figure 5C) that the PI3K-Akt signaling pathway, signaling pathways regulating pluripotency of stem cells, Wnt signaling pathway, and other signaling pathways were enriched. The hub genes *INSR*, *KIT*, *GNB4*, *GNB5*, *CSF1*, *ITGA2*, and *ITGAV* are all involved in the regulation of the PI3K-Akt signaling pathway. Combining the relationship between target regulation and expression level, a mammary-gland-development-related ceRNA regulatory network, including 11 circRNAs, 9 miRNAs, 37 genes, and 38 transcripts, was constructed (Figure 6 and Table S8). Transcripts corresponding to network hub genes were highly expressed during the DP (Figure 7C). It is worth noting that the hub gene *INSR* is located upstream of the PI3K-Akt signaling pathway (Figure S12). chi-miR-148b-3p can target and regulate *XM_018051136.1* (*INSR*), and their expression levels are significantly negatively correlated (cor = −0.89, *p*.adj < 0.01) (Figure 7D,F). chi-miR-148b-3p can also target and regulate circRNA *NC_030816.1:83819802|83825048*, and their expression levels are significantly negatively correlated (cor = −0.97, *p*.adj < 0.01). *NC_030816.1:83819802|83825048* was significantly positively correlated with *XM_018051136.1* (cor = 0.87, *p*.adj < 0.01), both of which were highly expressed during the DP (Figure 7A–C).

### 3.6. Analysis of circRNA–miRNA–mRNA ceRNA Regulatory Network Related to Substance Metabolism

A total of 39 genes related to substance metabolism and transport were screened, 11 of which were identified as the hub genes of the PPI network (Figure S13A). These genes are enriched in physiological processes, such as membrane lipid metabolic process, lipid transport, fatty acid metabolic process, and amino acid transmembrane transport (Figure S13B). KEGG results showed that the AMPK signaling pathway, glycosphingolipid biosynthesis-ganglio series, biosynthesis of amino acids, carbon metabolism, and other signaling pathways were enriched (Figure S13C). The hub genes *FASN*, *ELOVL5*, and *ACACA* are involved in the regulation of fatty acid metabolism (Figure S13D). *PEMT* and *LPIN1* are involved in the regulation of glycerophospholipid metabolism. Combining the relationship between target regulation and expression level, a substance metabolism-related ceRNA regulatory network, including 58 circRNAs, 36 miRNAs, 39 genes, and 48 transcripts, was constructed (Figure S14 and Table S9). Analysis of hub genes’ expression revealed that the *CD36* contained three transcripts, among which *XM_018046616.1* was highly expressed at LG, *XM_018046614.1* was highly expressed at LL, and *XM_018046615.1* was highly expressed at both LL and LG (Figure S15B). *CD36* is involved in the regulation of AMPK signaling pathway and cholesterol metabolism. In addition, chi-miR-130b-5p with *XM_018046614.1*, chi-miR-10a-5p with *XM_018046615.1*, chi-miR-125a-3p with *XM_018046615.1*, and chi-miR-29c-3p with *XM_018046616.1* has a targeted regulatory relationship predicted by bioinformatics, and their expression levels are negatively correlated. circRNAs *NC_030808.1:135270876|135279008*, *NC_030808.1:40225918|40234775*, *NC_030809.1:106427830|106442766*, and *NC_030812.1:112121725|112127445* can compete for the binding of chi-miR-130b-5p, which in turn may affect the expression of *CD36*. In addition, *FASN* (*NM_001285629.1*) and *FABP3* (*NM_001285701.1* and *XM_013971605.2*) were highly expressed at LL. *PEMT* is highly expressed at LG. *SLC7A5* and *SLC1A1* are involved in the amino-acid-transmembrane-transport process, and they are highly expressed at LL. In addition, only 3 hub gene transcripts were highly expressed during the DP, and the expression levels of the other 12 hub gene transcripts were decreased during the DP. Similarly, only three circRNAs were highly expressed during the DP, while other circRNAs were down-regulated during the DP (Figure S15A). This indicated that circRNAs and mRNAs related to substance metabolism were actively expressed at LL and LG. They are involved in the synthesis and metabolism of lipids, carbohydrates, and proteins, thus preparing for a new round of lactation.

### 3.7. Analysis of Immune-Related circRNA–miRNA–mRNA ceRNA Regulatory Network

A total of 22 genes related to mammary gland immunity were screened, 7 of which were identified as hub genes of the PPI network (Figure S16A). These genes are enriched in mononuclear cell differentiation, lymphocyte differentiation, cell activation involved in immune response, B cell activation, and other physiological processes. KEGG results showed (Figure S16C) that the B-cell-receptor signaling pathway, Fc gamma R-mediated phagocytosis, NF-kappa B signaling pathway, chemokine signaling pathway, and other signaling pathways were enriched. Combined with the targeted regulatory relationship and expression, an immune-related ceRNA network, including 46 circRNAs, 22 genes, 26 transcripts, and 21 miRNAs, was constructed (Figure S17). The hub gene *CASP8* was highly expressed in LG, and the other seven hub genes were highly expressed in the DP. Hub genes *BTK*, *INPP5D*, and *RAF1* are involved in the regulation of B-cell-receptor signaling pathway, Fc gamma R-mediated phagocytosis, and other signaling pathways. *BTK*, *CXCL12*, and *PRKCB* participate in the regulation of NF-kappa B signaling pathway. *CXCL12*, *RAF1*, and *PRKCB* participate in the regulation of the chemokine signaling pathway. The hub gene *CASP8* was highly expressed at LG, and the other seven hub genes were highly expressed in the DP (Figure S18). Hub genes *BTK*, *INPP5D*, and *RAF1* are involved in the regulation of the B-cell-receptor signaling pathway, Fc gamma R-mediated phagocytosis, and other signaling pathways. *BTK*, *CXCL12*, and *PRKCB* participate in the regulation of the NF-kappa B signaling pathway. *CXCL12*, *RAF1*, and *PRKCB* participate in the regulation of the chemokine signaling pathway. In addition, it was found that chi-miR-30b-5p can target and regulate *BTK*. *NC_030810.1:84946448|84965332*, *NC_030815.1:71328046|71344116*, *NC_030816.1:19670043|19696979*, *NC_030816.1: 26231856|26242268*, and other circRNAs can compete for binding to chi-miR-30b-5p through bioinformatics predictions. Chi-miR-29c-3p can target and regulate *CASP8*. *NC_030808.1:142388405|142433828*, *NC_030812.1:12090221|12092129*, NC_030814.1:56550226|56581054, and *NC_030814.1:75848324|75868791*, etc., can compete for the binding of chi-miR-29c-3p. chi-miR-660 can target and regulate *CXCL12*. *NC_030821.1:16648181|16649027* can compete for binding to chi-miR-660.

### 3.8. Analysis of circRNA–miRNA–mRNA ceRNA Regulatory Network Related to Mammary Cell Apoptosis

A total of 27 genes related to apoptosis were screened, and 7 genes were identified as the hub genes of the PPI network (Figure S19A). These genes were enriched to the leukocyte apoptosis process, lymphocyte apoptosis process, B-cell-apoptosis process, and other physiological processes (Figure S19B). KEGG results showed (Figure S19D) that apoptosis, the JAK-STAT signaling pathway, the FoxO signaling pathway, the PI3K-Akt signaling pathway, the TNF signaling pathway, and other signaling pathways were significantly enriched (FDR < 0.05). Combined with the targeted regulatory relationship, an apoptosis-related ceRNA network, including 26 circRNAs, 27 genes, 30 transcripts, and 12 miRNAs, was constructed (Figure S20). Hub genes *MAP3K5*, *IL7R*, *BTK*, *IKBKG*, *TNFRSF1A*, and *IL2RA* were highly expressed during the DP (Figure S21). *CSF2RB* was low expressed during the DP. In addition, it was found through bioinformatics prediction that chi-miR-148a-3p can target and regulate *TNFRSF1A* (*XM_018048606.1*), and *NC_030818.1:70611932|70614030* can compete for binding to chi-miR-148a-3p. chi-miR-30b-5p can target and regulate *MAP3K5* (*XM_005684842.3*). *NC_030816.1:19670043|19696979*, *NC_030820.1:37909806|37923941*, and *NC_030821.1:56966260|56986994* can compete with chi-miR-30b-5p for binding. Hub genes *MAP3K5*, *IL7R*, *BTK*, *IKBKG*, *TNFRSF1A*, and *IL2RA* were highly expressed during the DP. *CSF2RB* was low expressed during the DP. In addition, it was found through bioinformatics prediction that chi-miR-148a-3p can target and regulate *TNFRSF1A* (*XM_018048606.1*), and *NC_030818.1:70611932|70614030* can compete for binding to chi-miR-148a-3p. chi-miR-30b-5p can target and regulate *MAP3K5* (*XM_005684842.3*); *NC_030816.1:19670043|19696979*, *NC_030820.1:37909806|37923941*, and *NC_030821.1:56966260|56986994* could compete with chi-miR-30b-5p for binding. In addition, a total of six genes, including *IL2RA*, *IL7R*, *CSF2RB*, *JAK3*, *AKT3*, and *IL3RA*, were enriched in the JAK-STAT signaling pathway. Among them, *JAK3* (*XM_018051403.1*) was targeted and regulated by chi-miR-30b-5p. *NC_030821.1:56966260|56986994* and *NC_030831.1:54852737|54883065* can compete for binding to chi-miR-30b-5p.

## 4. Discussion

circRNAs are a new class of endogenous non-coding RNA molecules formed by covalent bonds, and they have been shown to play an important role in various physiological activities of the mammary gland [21]. However, there are few reports on the role and regulatory mechanism of circRNAs in the involution and remodeling of the non-lactating mammary gland. The mammary gland is known to undergo involution and cellular remodeling from LL to LG, which is accompanied by multiple physiological processes, such as apoptosis, proliferation, differentiation, milk synthesis, and secretion [3,5]. Therefore, this study explores the expression characteristics of circRNAs and the regulatory relationship between circRNAs and miRNAs from LL to LG, which is crucial for an in-depth understanding of physiological changes in mammary gland involution and cell remodeling.

This study found that circRNAs were expressed in mammary gland tissues at different developmental stages with time specificity (Figure 1C and Figure 3A). This is similar to the differential expression of circRNAs in the mammary glands of goats [18], sheep [20], and cattle [21] at different developmental stages. This study also found that the number of circRNAs expressed in mammary gland tissues during the dry period was higher than that at late lactation and late gestation (Figure 1C). However, the results of this study on goat mammary glands differ from the above-mentioned circRNAs in sheep mammary glands. This is related to the selection of mammary tissues at different developmental stages. In addition, studies have found that the number of circRNAs in adult mammary gland tissues is higher than that in other adult tissues (colon, heart, kidney, liver, lung, and stomach) and other glandular tissues (adrenal, pancreas, and thyroid), demonstrating tissue-specific expression of circRNAs [24].

circRNAs have similar structural features as regulators of gene expression. The number of circRNAs derived from exons was the largest (accounting for 90.6% of all circRNAs) in this study (Figure 1D). This is consistent with the circRNA features previously found in the mammary gland tissues of goats [18], sheep [20], cattle [21], mice [23], and other animals. The length of circRNAs identified in goat-mammary-gland tissues is mainly distributed in the range of 200 to 400 bp (Figure S2). This is similar to the length distribution of circRNAs in Holstein cow’s mammary gland tissues [22] and goat skin tissues [48]. In addition, this study also found that Chromosome 1, Chromosome 3, and Chromosome 10 produced more circRNAs than other chromosomes. This is similar to the chromosomal distribution of circRNAs identified in goat skeletal muscle [49].

The functions of circRNAs’ source genes are rich and involve various physiological processes and signaling pathways (Figure 2 and Figures S3–S6). It is known that in mammary epithelial cells, the activation and inactivation of the PI3K-Akt signaling pathway, MAPK signaling pathway, and ErbB signaling pathway affect most of metabolic mechanisms and biological pathways, including gene transcription, cell proliferation activity, cell apoptosis, cell cycle, etc. [13,50,51]. This study significantly enriched circRNAs’ source genes in the above three pathways. In addition, circRNAs’ source genes in bovine mammary epithelial cells were also enriched in signaling pathways related to cell growth, apoptosis, and immunity [27]. Interestingly, four casein-encoding genes (*CSN1S1*, *CSN1S2*, *CSN2*, and *CSN3*) and alpha-lactalbumin-encoding gene (*LALBA*) produced circular RNAs in mammary gland tissues at LL and LG, but their expression levels were not expressed during the DP (Table S2). In addition, circRNAs with higher expression in LL and LG came from *CSN1S1* and *CSN1S2*, which may be related to the active expression of genes encoding milk protein and casein during lactation. Similarly, genes encoding casein and lactalbumin were also detected to produce circRNAs in the mammary gland tissues of sheep [52] and cattle [21,27] during peak lactation. This suggests that the circRNAs discovered in this study may have the function of regulating milk synthesis and stimulating mammary gland growth and development.

Accumulating evidence suggests that circRNAs function as miRNA sponges, preventing miRNAs from binding to target genes, thereby regulating mammary gland development and lactation physiological processes [20,22,53,54]. In this study, circRNA–miRNA–mRNA ceRNA networks related to mammary gland development, cell apoptosis, substance metabolism, and immune function were constructed (Figure 6 and Figures S14, S17 and S20). *INSR* was identified as a hub gene in the mammary-gland-development ceRNA network. The *INSR*-encoded protein could regulate the uptake and release of glucose, as well as the synthesis and storage of carbohydrates, lipids, and proteins [55]. In addition, the insulin receptor plays an important role in the differentiation of mammary gland secretory cells and can regulate the growth and differentiation of mammary gland cells by affecting the PI3K-AKT signaling pathway and the Ras-MAPK signaling pathway [55,56,57]. Furthermore, miR-148b-3p has been reported to regulate breast cancer cell growth and serve as a potential marker of breast cancer [58]. Therefore, the ceRNA relationship of *NC_030816.1:83819802|83825048* and *NC_030829.1:30515090|30520814-*chi-miR-148b-3p-*INSR* may regulate the growth state of goat-mammary-gland cells.

One of the characteristics of mammary gland involution is extensive apoptosis of mammary cells [59]. Previous studies have demonstrated that circRNAs are involved in the regulation of mammary cell apoptosis. For example, the knockdown of hsa_circ_0001982 inhibits breast cancer cell proliferation and invasion and induces apoptosis by targeting miR-143, providing new insights into the pathogenesis of breast cancer [60]. Furthermore, knockdown of circ-ABCB10 inhibits breast cancer cell proliferation and increased apoptosis, revealing an important regulatory role of circ-ABCB10 through sponging miR-1271 [61]. In this study, an apoptosis-related ceRNA network was constructed (Figure S20 and Table S11). Among them, these apoptosis-related genes participate in the regulation of apoptosis, the JAK-STAT signaling pathway, the FoxO signaling pathway, the PI3K-Akt signaling pathway, the TNF signaling pathway, and other signaling pathways (Figure S19). It has been reported that miR-103a-3p and miR-107-3p are involved in the regulation of apoptosis [62,63]. Both chi-miR-103-3p and chi-miR-107-3p are in the ceRNA network related to apoptosis. The above supports the notion that circRNAs can affect the apoptosis process of mammary gland cells during involution by competitively binding with miRNAs.

Secretion composition of mammary gland changes during involution [10]. The mammary epithelium is the factory that synthesizes and processes milk fat, protein, and carbohydrates. In this study, circRNAs related to substance metabolism were screened and a circRNAs–miRNAs–mRNAs ceRNA regulatory network related to metabolism was constructed (Figure S14). Among them, the ceRNA relationship of *NC_030808.1:40225918|40234775*, *NC_030809.1:106427830|106442766*, and *NC_030812.1:113584493|113589629*-chi-miR-130b-5p-*FAPB3* may be related to lipid metabolism. On the one hand, *FABP3* is considered to be involved in the uptake of long-chain fatty acids, intracellular metabolism, and material transportation; on the other hand, it can also inhibit the growth of mammary epithelial cells [64]. This study found that *FABP3* was highly expressed at LL (Figure S15B), which may be related to the decline in the ability of mammary cells to synthesize and secrete substances and cell apoptosis at the end of lactation. miR-130b-5p has regulatory effects on lipid accumulation and metabolism in mouse liver [65]. The above circRNAs may affect the process of mammary gland lipid synthesis by combining with miR-130b-5p. In addition, *SLC7A5*, *SLC1A1*, *SLC38A1*, *SLC7A7*, and *SLC7A4* are involved in amino acid transmembrane transport. In this study, a circRNA–miRNA–mRNA ceRNA network related to the above amino acid synthesis and transport was constructed. *B3GALT4* and *B3GNT5* are involved in carbohydrate metabolism [66]. chi-miR-30b-5p can target and regulate *B3GNT5*, and chi-miR-423-3p can target and regulate *B3GALT4*. *NC_030810.1:84946448|84965332* can compete with chi-miR-30b-5p, and *NC_030815.1:76944795|76962878* can compete with chi-miR-423-3p. Research on circRNAs that can regulate the metabolism of substances in the mammary gland has been extensively conducted on dairy goats cows [22], and sheep [52]. In conclusion, it is illustrated that circRNAs are involved in the regulation of physiological processes, such as the synthesis and metabolism of lipids, proteins, carbohydrates, and other substances during the mammary involution process.

The presence of inflammatory cells in the involutional mammary gland helps to remove apoptotic cells and residual milk, thereby promoting mammary gland remodeling [9]. The immune system is a complex network that maintains the physiological balance and stability of the body’s internal environment. There has been some evidence that circRNAs are directly involved in immune regulation. circRNAs can competitively bind double-stranded RNA-activated protein kinase (PKR), thereby broadly regulating cellular immune signaling pathways [67]. circZC3H4 has been shown to be involved in SiO_2_-induced macrophage activation through the HECTD1/ZC3H12A-dependent ubiquitin-proteasome pathway [68]. Furthermore, cadmium promotes apoptosis and inflammation in mammary epithelial cells of bovine and mice through the circ08409/miR-133a/TGFB2 axis [29]. In this study, immune-related circRNAs were identified in goat-mammary-gland tissues (Figures S16 and S17 and Table S10). *NC_030808.1:142388405|142433828*, *NC_030812.1:12090221|12092129*, *NC_030814.1:56550226|56581054*, *NC_030814.1:75848324|75868791*, and other circRNAs can compete for binding to chi-miR-29c-3p. Meanwhile, a regulatory loop between miR-29c-3p and gene *Myc* exists in cancer cells to counteract the cytotoxicity of natural killer cells [69]. Other studies identified the AC005154.6/hsa-miR-29c-3p/*CCNL2* axis as a novel prognostic biomarker associated with immune infiltration in prostate cancer [70]. In addition, phagocytosis-related genes, such as *CLEC7A*, *CD36*, and *DOCK2*, were also identified in the immune-related ceRNA network. This plays an important role in removing apoptotic cells and residual milk in involutional mammary gland tissue [71], indicating that the immune-related circRNAs discovered in this study may play a regulatory role in maintaining the homeostasis of the mammary gland during involution and promoting mammary tissue remodeling.

This study also has some limitations. First, as explained in our previous work [13,30], the ability to detect differentially expressed circRNAs was limited due to small sample size (three replicates in each stage). Second, our analysis excluded low-expression transcripts (FPKM < 2), which might overlook the role of low-expressed circRNAs in mammary cell involution and remodeling. Furthermore, the study only assessed three-time points (long time intervals) during mammary gland involution. Although differences in mammary gland function and structure have been found, mammary gland involution and remodeling is a continuous process [4]. More sampling and sequencing time points may help to better explain the molecular mechanisms behind the changes in this process, as well as provide a clearer and more accurate interpretation of the spatiotemporal characteristics of various physiological activities during mammary gland involution.

## 5. Conclusions

In this study, a total of 11,756 circRNAs were identified in the non-lactating goat-mammary-gland group by Illumina sequencing. Among them, 218 circRNAs were differentially expressed in non-lactating mammary glands, with the highest number of circRNAs specifically expressed in the DP and the least number of circRNAs specifically expressed in LL. On the one hand, this shows that the expression of circRNAs changes at the transcriptional level during mammary gland involution; on the other hand, it indicates the temporal specificity of circRNAs expressed in mammary gland tissues at different developmental stages. GO functional analysis of cicrRNA-derived genes found that GO terms, such as histone modification, regulation of GTPase activity, positive regulation of organelle organization, and establishment or maintenance of cell polarity, were significantly enriched, which reflected the diversity of circRNAs’ source gene functions. Both genes that encode lactalbumin and casein can produce circRNAs, indicating that circRNAs may regulate milk protein synthesis. In addition, this study also constructed circRNA–miRNA–mRNA ceRNA regulatory networks related to mammary gland development, immunity, substance metabolism, and cell apoptosis, indicating that circRNAs can affect physiological processes, such as cell apoptosis, growth, substance metabolism, tissue remodeling, and intercellular connection and communication. Taken together, these findings provide new insights into the role of circRNAs in regulating goat-mammary-gland involution and cellular remodeling.

## Data Availability

The data presented in this study are available on request.

## Supplementary Materials

The following supporting information can be downloaded at: https://www.mdpi.com/article/10.3390/biom13050860/s1. Figure S1. Analysis of the position of circRNAs in the chromosome and the number of circRNAs produced by each gene; Figure S2. Length distribution of circRNAs; Figure S3. GO functions annotation analysis of up-regulated and down-regulated circRNAs source genes in DP vs. LG and LL vs. LG; Figure S4. GO functions annotation analysis on the up/down-regulated genes targeting the differentially expressed circRNAs in LL vs. DP; Figure S5. GO functions annotation analysis on the up/down-regulated genes targeting the differentially expressed circRNAs in DP vs. LG; Figure S6. GO functions annotation analysis on the up/down-regulated genes targeting the differentially expressed circRNAs in LL vs. LG; Figure S7. Volcano maps of differentially expressed circRNAs in DP vs. LG and LL vs. LG. (A) and (B) respectively refer to DP vs. LG and LL vs. LG; Figure S8. Expression histogram of differentially expressed circRNAs at different stages of mammary gland development; Figure S9. Verify the expression of circRNAs by RTqPCR; Figure S10. circRNAs sequence information was verified by Sanger sequencing; Figure S11. Alignment results of 14 circRNAs sequences obtained by sanger sequencing with reference sequences of goat; Figure S12. A map of PI3K-Akt signaling pathway; Figure S13. Analysis of circRNA-miRNA-mRNA ceRNA regulatory network related to mammary gland substance metabolism; Figure S14. Analysis of circRNA-miRNA-mRNA ceRNA regulatory network related to mammary gland substance metabolism; Figure S15. Expression heat map of mammary gland substance metabolism-related circRNAs, mRNAs, and miRNAs; Figure S16. Analysis of immune-related circRNA-miRNA-mRNA ceRNA regulatory network in mammary gland; Figure S17. Analysis of circRNA-miRNA-mRNA ceRNA regulatory network related to mammary gland immunity; Figure S18. Expression heat map of mammary immune-related circRNAs, mRNAs, and miRNAs; Figure S19. Analysis of mammary cell apoptosis-related circRNA-miRNA-mRNA ceRNA regulatory network; Figure S20. Analysis of circRNA-miRNA-mRNA ceRNA regulatory network related to mammary cell apoptosis; Figure S21. Expression heat map of mammary cell apoptosis-related circRNAs, mRNAs and miRNAs; Table S1. Basic statistics of sequencing results; Table S2. Source genes and classification of circRNAs; Table S3. GO functional annotation and KEGG pathway enrichment analysis of circRNA source genes; Table S4. Results of circRNAs differential expression analysis; Table S5. Primer sequences of circRNAs; Table S6. The prediction results of the targeting relationship between miRNAs and circRNAs or miRNAs and mRNAs; Table S7. Clustering and functional analysis of differentially expressed circRNAs; Table S8. Analysis of circRNA-miRNA-mRNA ceRNA regulatory network related to mammary gland development; Table S9. Analysis of circRNA-miRNA-mRNA ceRNA regulatory network related to substance metabolism; Table S10. Analysis of immune-related circRNA-miRNA-mRNA ceRNA regulatory network in mammary gland; Table S11. Analysis of mammary cell apoptosis-related circRNA-miRNA-mRNA ceRNA regulatory network.
