# Supplementary material for: Identification and Characterization of circRNAs in Non-Lactating Dairy Goat Mammary Glands Reveal Their Regulatory Role in Mammary Cell Involution and Remodeling
Source: Biomolecules. 2023 May 18;13(5):860. doi: 10.3390/biom13050860 (PMC10216389; doi:10.3390/biom13050860)
Supplement: Supplementary file 1 [file biomolecules-13-00860-s001.zip › Supplementary materials/Supplementary Figures.pdf]

# Supplementary Figures

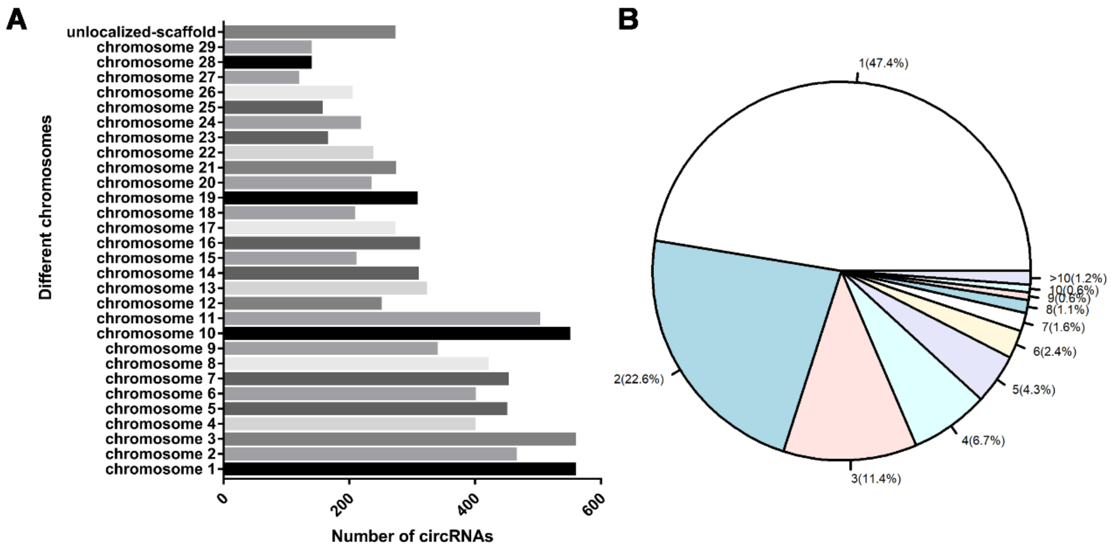


**Figure S1.** Analysis of the position of circRNAs in the chromosome and the number of circRNAs produced by each gene. (**A**) The number of circRNAs in the chromosome; (**B**) The number of circRNAs produced by each gene.

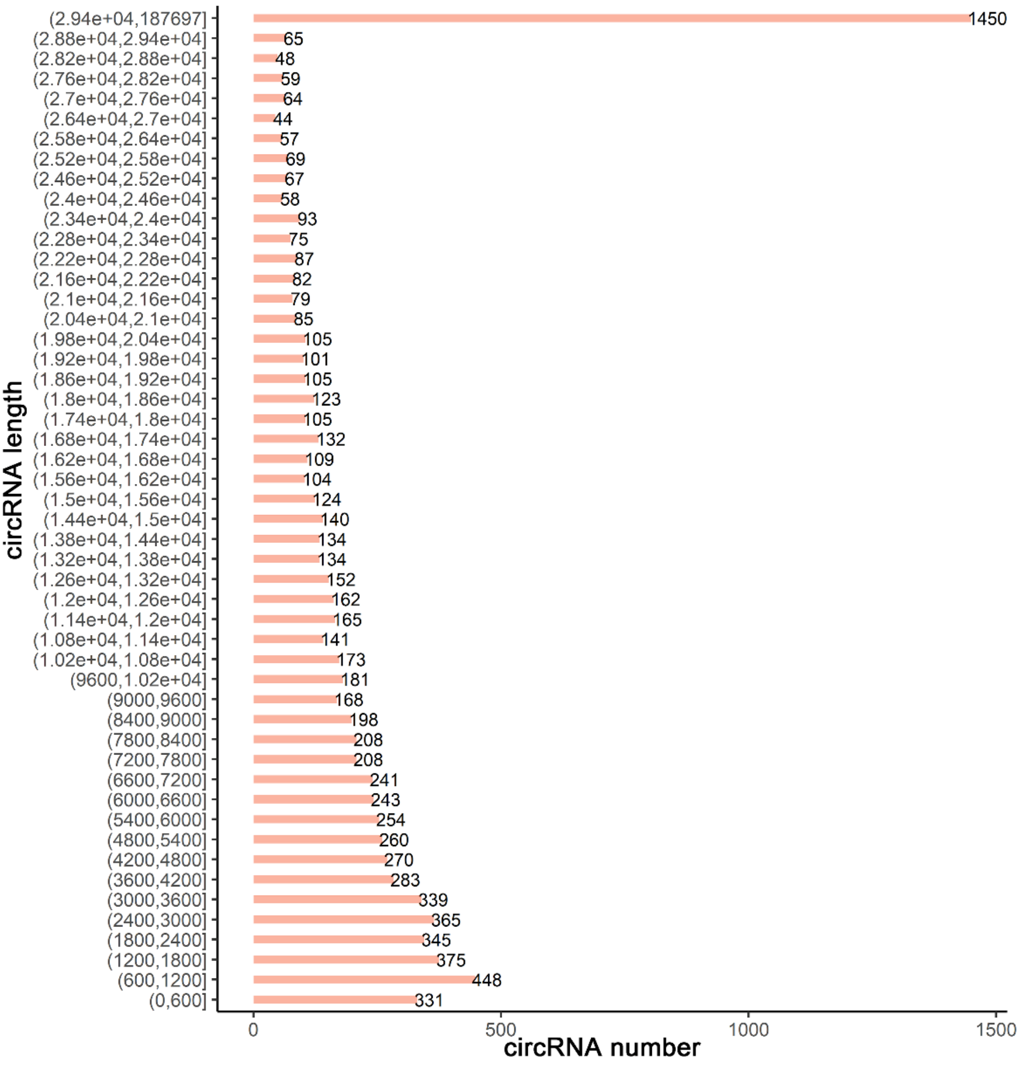


**Figure S2.** Length distribution of circRNAs.

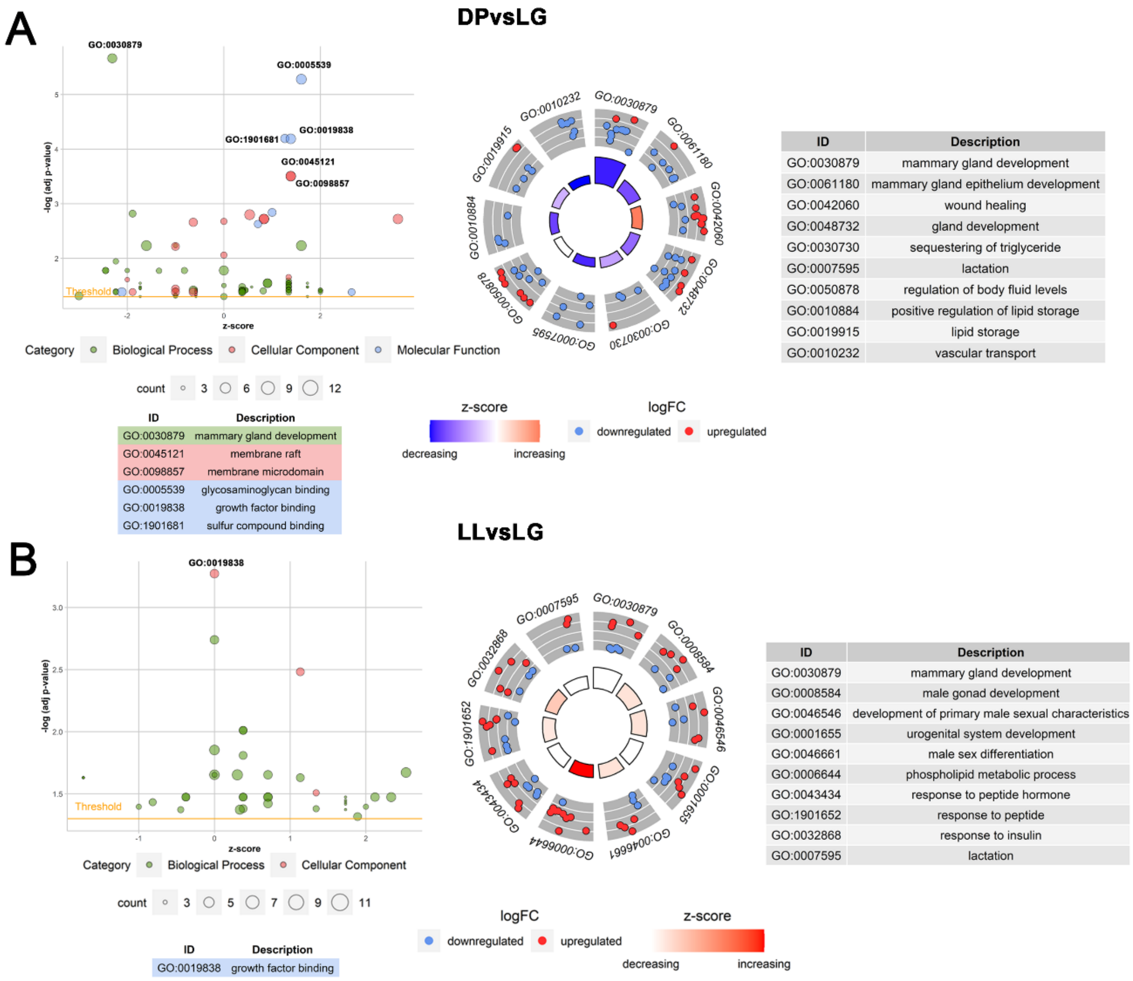


**Figure S3.** GO functions annotation analysis of up-regulated and down-regulated circRNAs source genes in DP vs. LG and LL vs. LG. LL: late lactation, DP: dry period, and LG: late gestation.

A

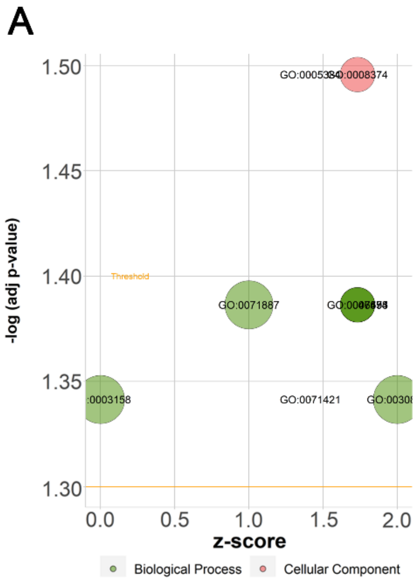


| ID | Description |
|---|---|
| GO:0006654 | phosphatidic acid biosynthetic process |
| GO:0046473 | phosphatidic acid metabolic process |
| GO:0071887 | leukocyte apoptotic process |
| GO:0007595 | lactation |
| GO:0071421 | manganese ion transmembrane transport |
| GO:0003158 | endothelium development |
| GO:0030879 | mammary gland development |
| GO:0005384 | manganese ion transmembrane transporter activity |
| GO:0008374 | O-acyltransferase activity |

B

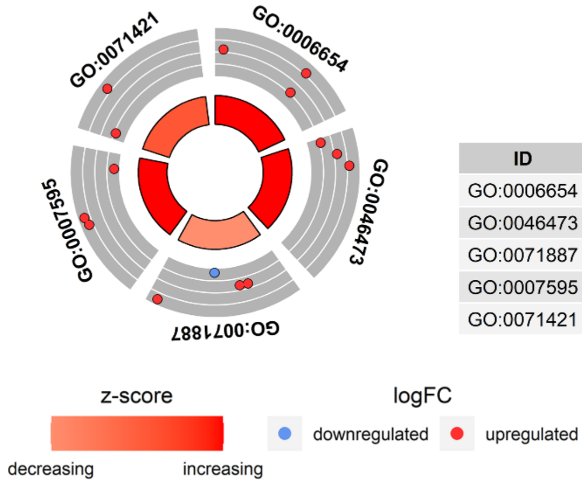


| ID | Description |
|---|---|
| GO:0006654 | phosphatidic acid biosynthetic process |
| GO:0046473 | phosphatidic acid metabolic process |
| GO:0071887 | leukocyte apoptotic process |
| GO:0007595 | lactation |
| GO:0071421 | manganese ion transmembrane transport |

**Figure S4.** GO functions annotation analysis on the up/down-regulated genes targeting the differentially expressed circRNAs in LL vs. DP. LL: late lactation, DP: dry period.

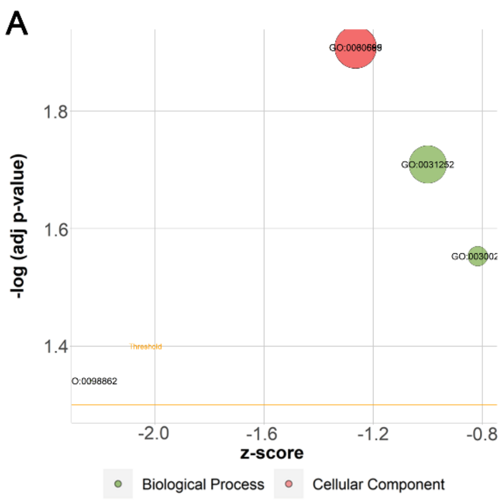


| ID | Description |
|---|---|
| GO:0031252 | cell leading edge |
| GO:0030027 | lamellipodium |
| GO:0098862 | cluster of actin-based cell projections |
| GO:0030695 | GTPase regulator activity |
| GO:0060589 | nucleoside-triphosphatase regulator activity |

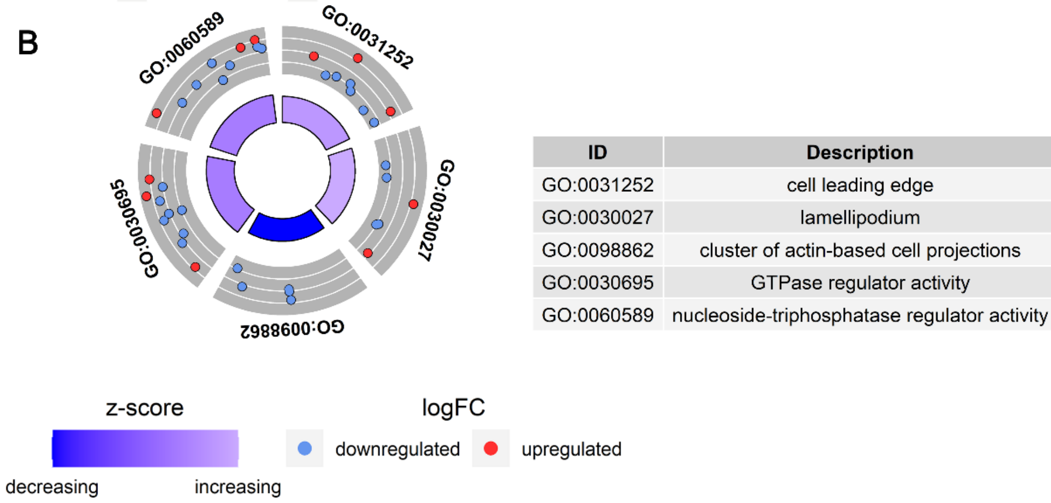


| ID | Description |
|---|---|
| GO:0031252 | cell leading edge |
| GO:0030027 | lamellipodium |
| GO:0098862 | cluster of actin-based cell projections |
| GO:0030695 | GTPase regulator activity |
| GO:0060589 | nucleoside-triphosphatase regulator activity |

**Figure S5.** GO functions annotation analysis on the up/down-regulated genes targeting the differentially expressed circRNAs in DP vs. LG. LG: late gestation, DP: dry period.

A

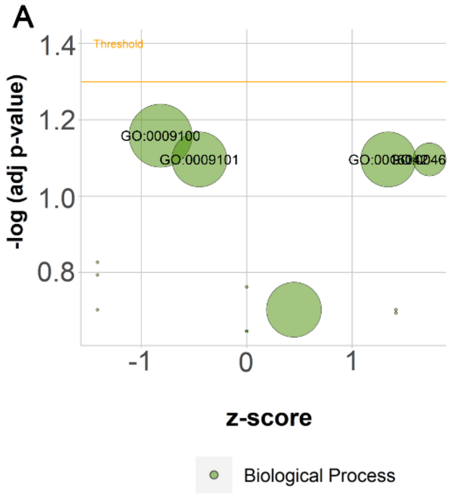


| ID | Description |
|---|---|
| GO:0009100 | glycoprotein metabolic process |
| GO:0046503 | glycerolipid catabolic process |
| GO:0009101 | glycoprotein biosynthetic process |
| GO:0016042 | lipid catabolic process |

B

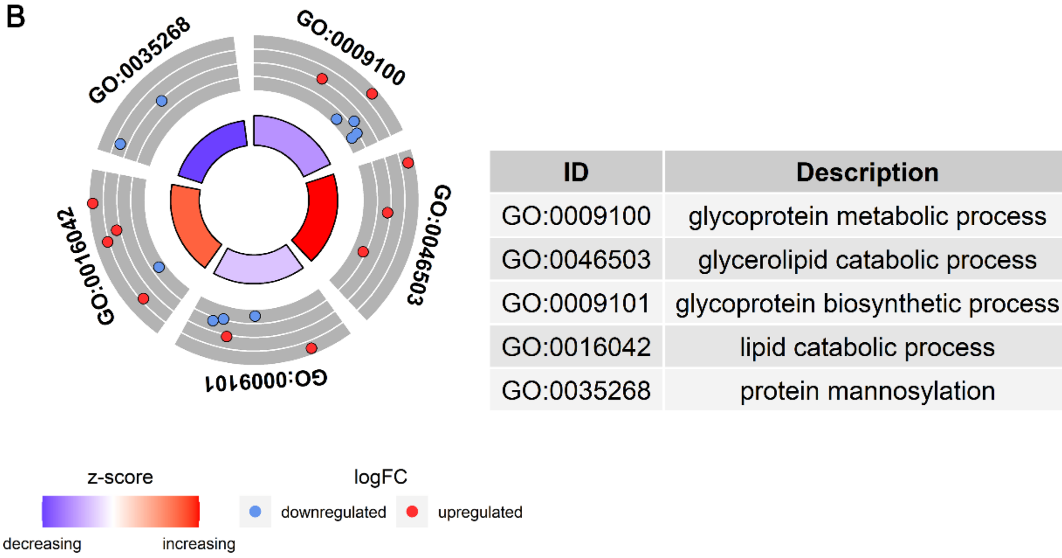


| ID | Description |
|---|---|
| GO:0009100 | glycoprotein metabolic process |
| GO:0046503 | glycerolipid catabolic process |
| GO:0009101 | glycoprotein biosynthetic process |
| GO:0016042 | lipid catabolic process |
| GO:0035268 | protein mannosylation |

**Figure S6.** GO functions annotation analysis on the up/down-regulated genes targeting the differentially expressed circRNAs in LL vs. LG. LL: late lactation, LG: late gestation.

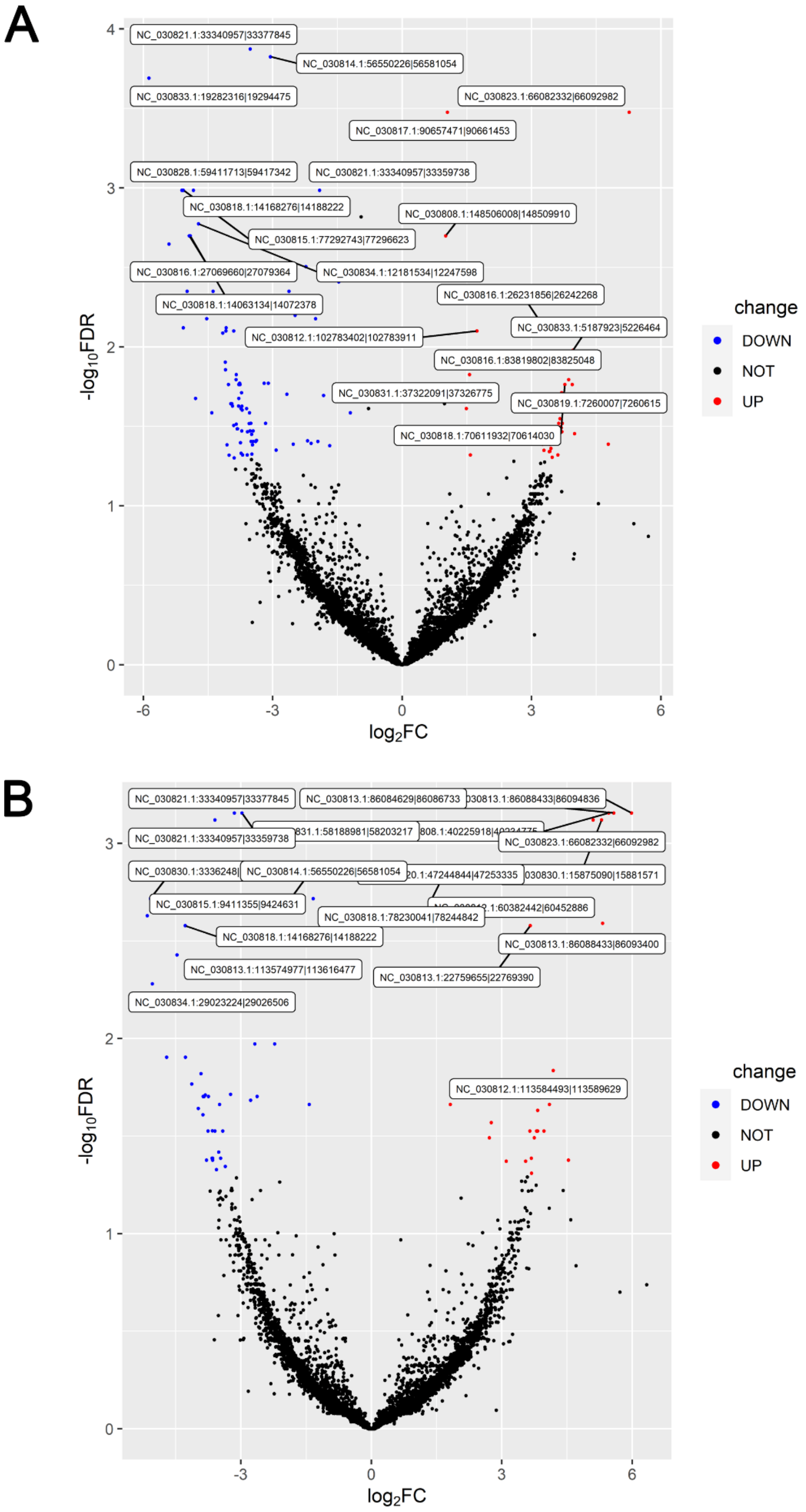


**Figure S7.** Volcano maps of differentially expressed circRNAs in DP vs. LG and LL vs. LG. (**A**) and (**B**) respectively refer to DP vs. LG and LL vs. LG. LL: late lactation, DP: dry period, and LG: late gestation.

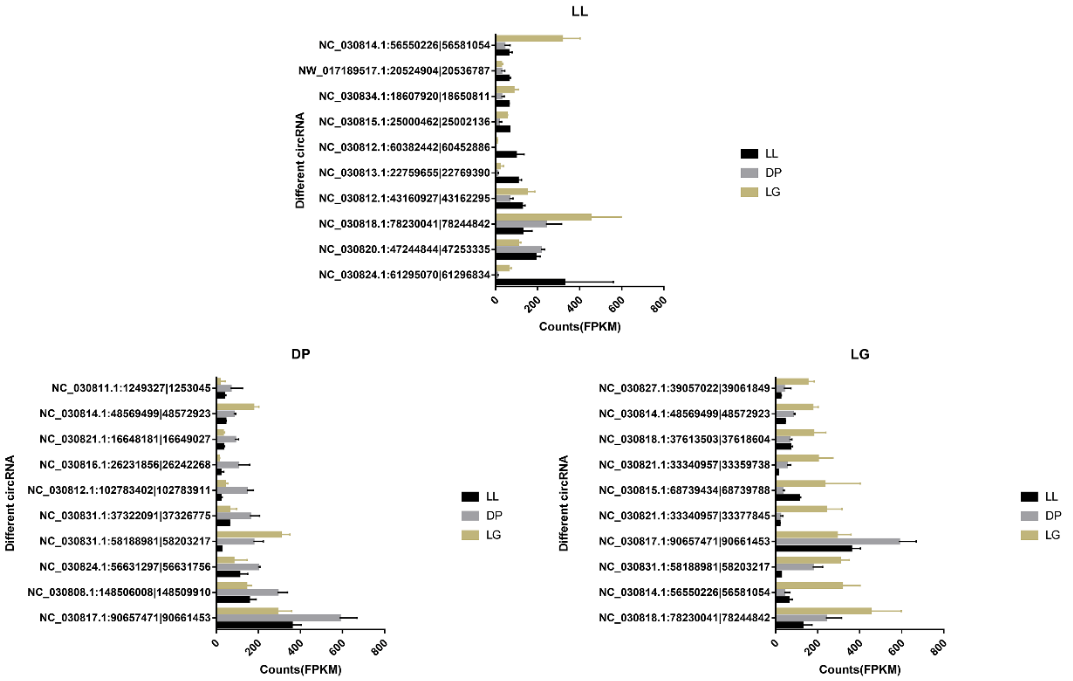


**Figure S8.** Expression histogram of differentially expressed circRNAs at different stages of mammary gland development. The differentially expressed circRNAs were sorted according to the expression level at each developmental stage, and the top 10 differentially expressed circRNAs at each developmental stage (LL, DP, and LG) were selected and displayed using a histogram. LL: late lactation, DP: dry period, and LG: late gestation.

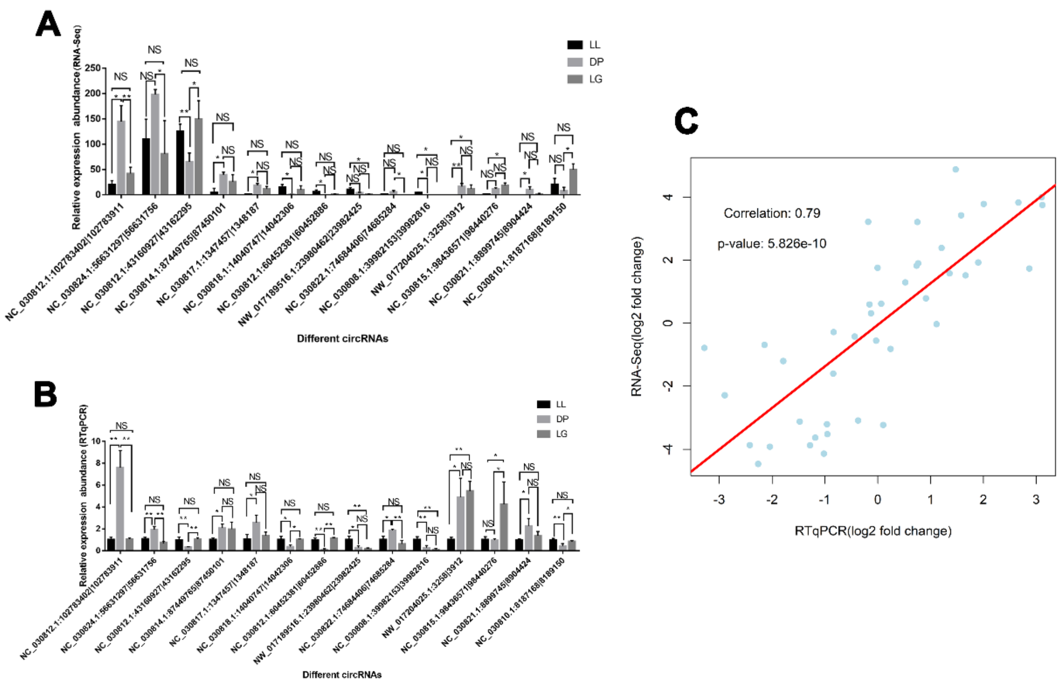


**Figure S9.** Verify the expression of circRNAs by RTqPCR. (**A**) The expression level of circRNAs detected by transcriptome sequencing; (**B**) The expression level of circRNAs detected by RTqPCR. (**C**) Log2 fold change correlation between RNA-Seq and RTqPCR. LL: late lactation, DP: dry period, and LG: late gestation. * indicates *P*-value < 0.05, ** indicate *P*-value < 0.01, and NS indicate Not Significant.

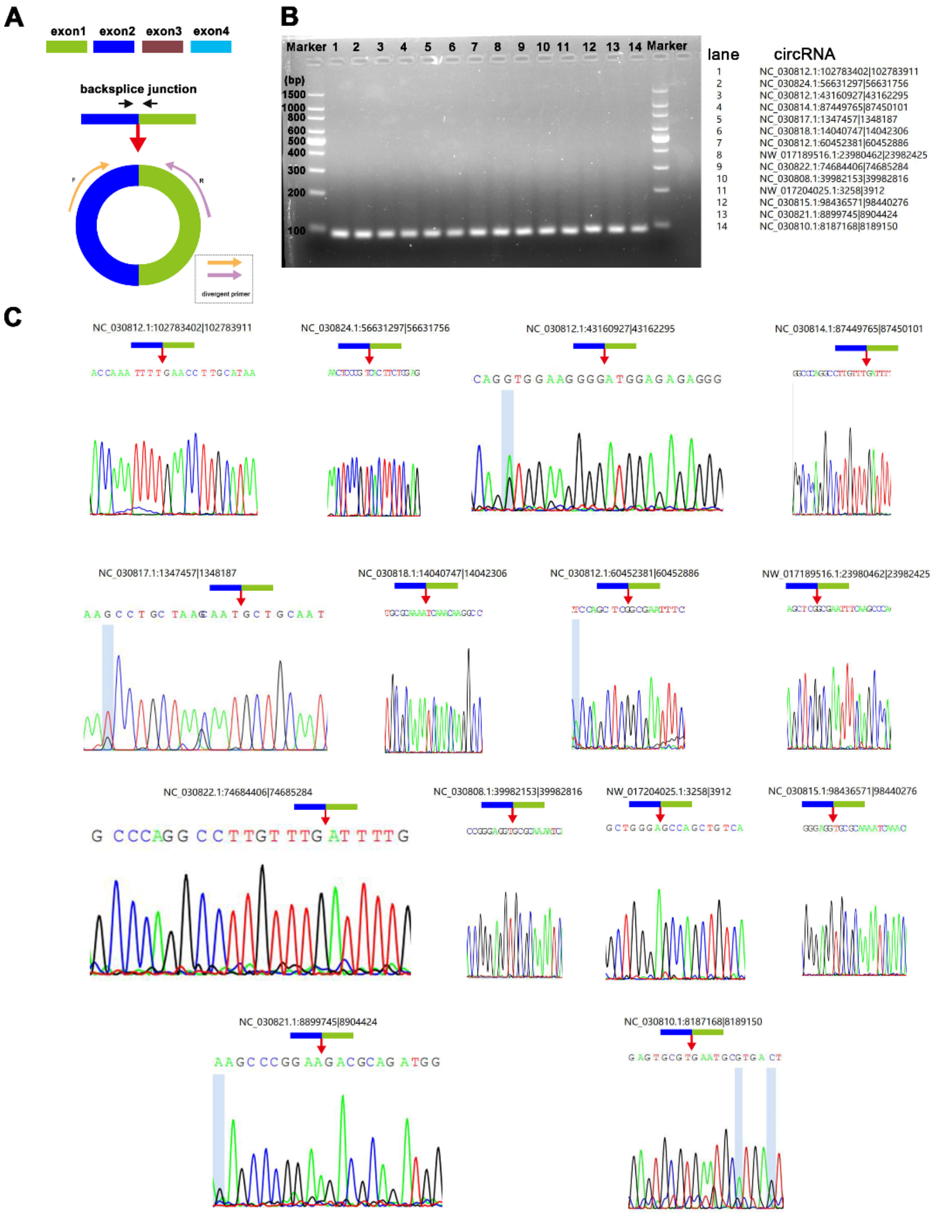


**Figure S10.** circRNAs sequence information was verified by Sanger sequencing. (**A**) A design schema of circRNAs divergent primers; (**B**) Agarose electrophoresis gel images of 14 circRNAs amplification products; (**C**) The looping site sequences of 14 circRNAs.

**NC_030808.1:39982153|39982816**

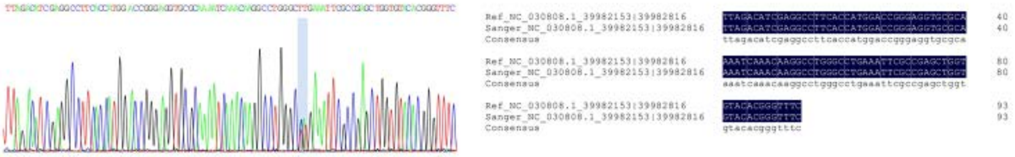

**NC_030810.1:8187168|8189150**

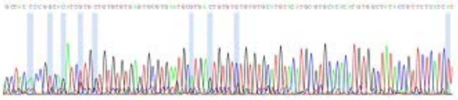

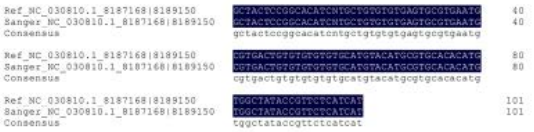

**NC_030812.1:43160927|43162295**

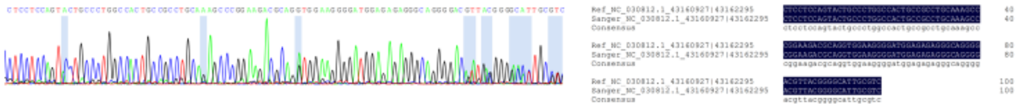

**NC_030812.1:60452381|60452886**

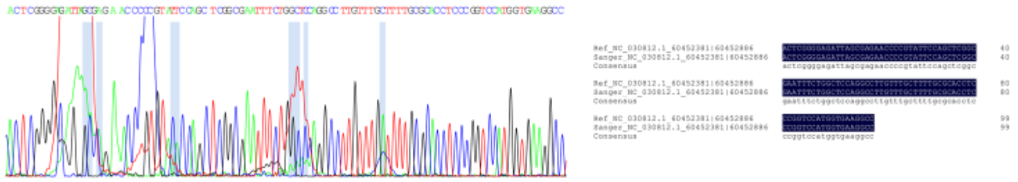

**NC_030812.1:102783402|102783911**

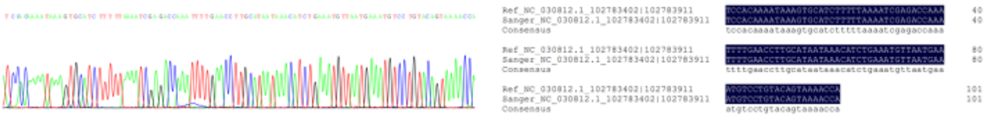

**NC_030814.1:87449765|87450101**

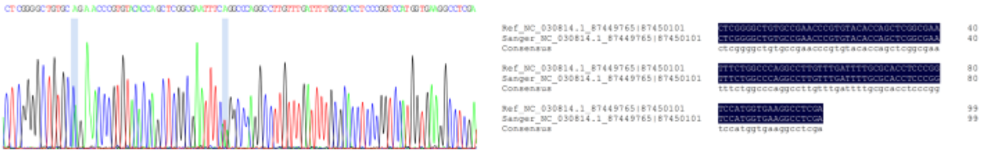

**NC_030815.1:98436571|98440276**

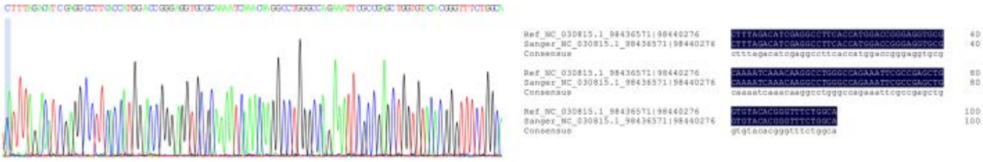

**NC_030817.1:1347457|1348187**

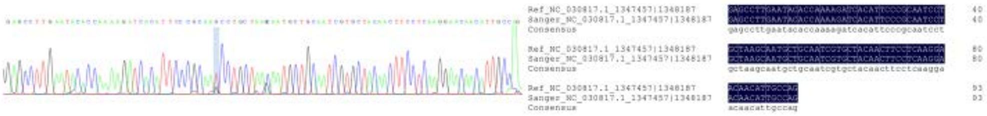

**NC_030818.1:14040747|14042306**

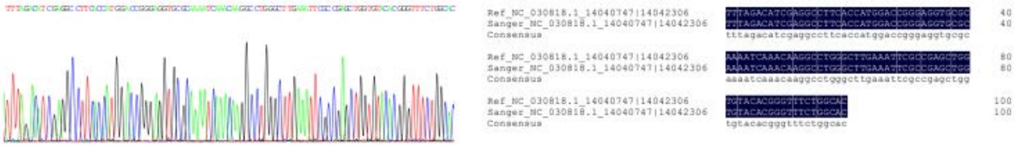


**NC_030821.1:8899745|8904424**

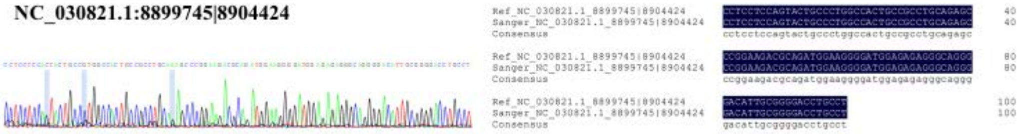


**NC_030822.1:74684406|74685284**

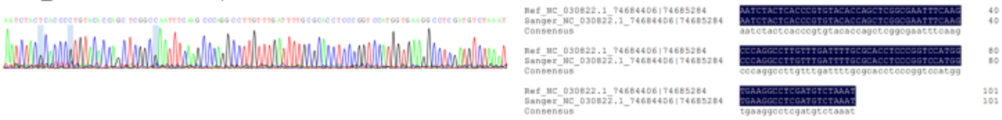


**NC_030824.1:56631297|56631756**

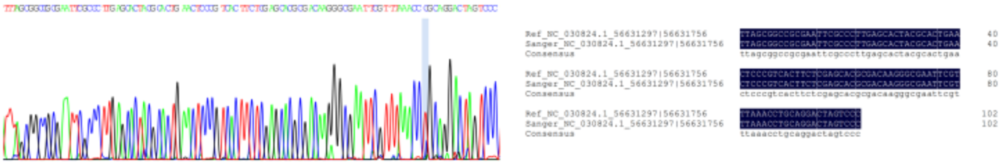

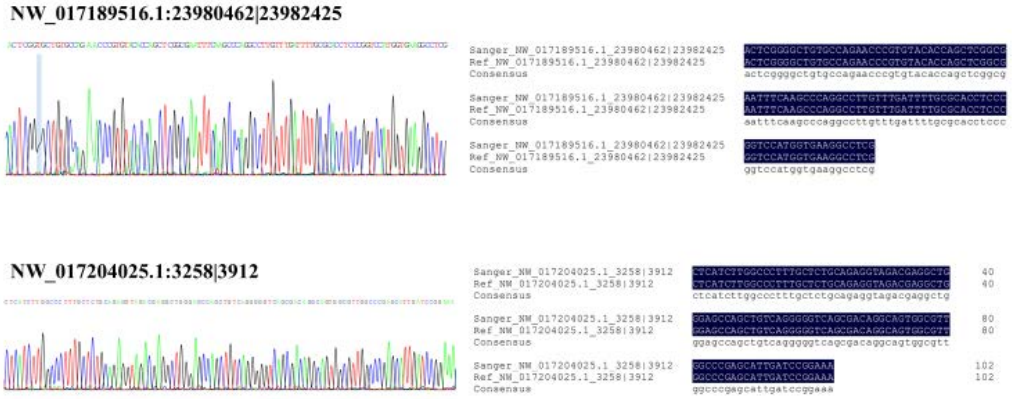


**Figure S11.** Alignment results of 14 circRNAs sequences obtained by sanger sequencing with reference sequences of goat.

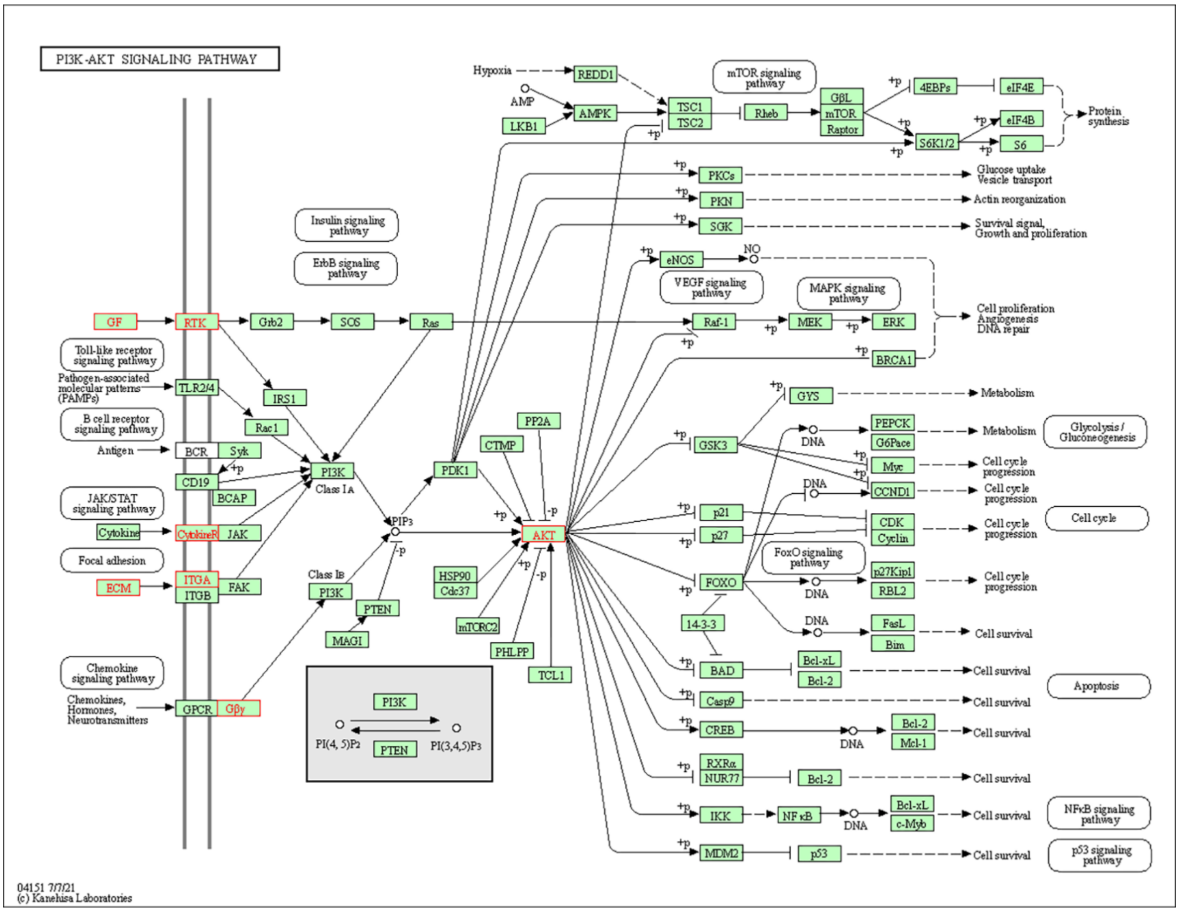

**Figure S12.** A map of PI3K-Akt signaling pathway.

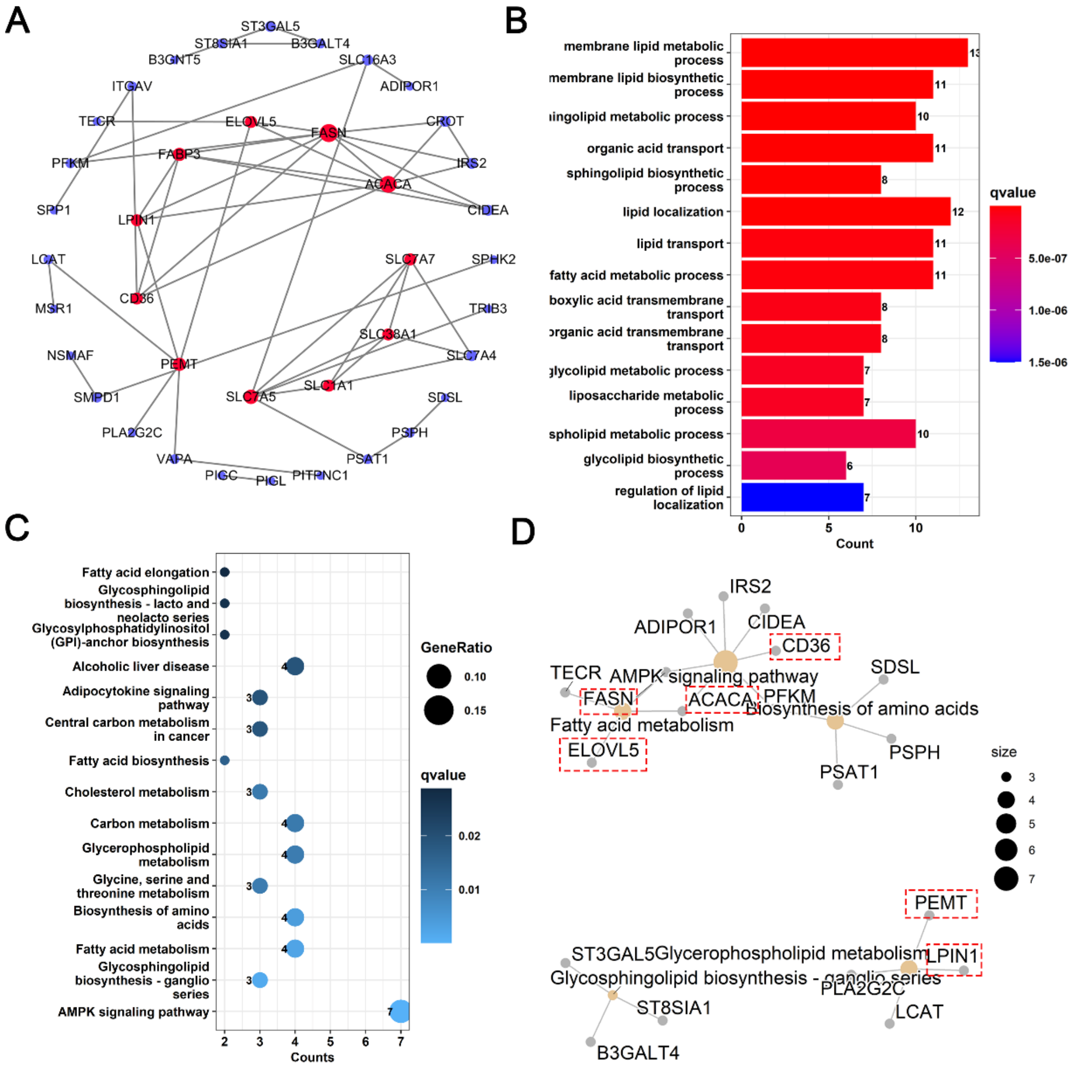


**Figure S13.** Analysis of circRNA-miRNA-mRNA ceRNA regulatory network related to mammary gland substance metabolism. (**A**) PPI network analysis of genes related to mammary gland substance metabolism; (**B**) GO analysis of genes related to mammary gland substance metabolism; (**C**) KEGG analysis of genes related to mammary gland substance metabolism; (**D**) Relationship between KEGG pathways and genes.

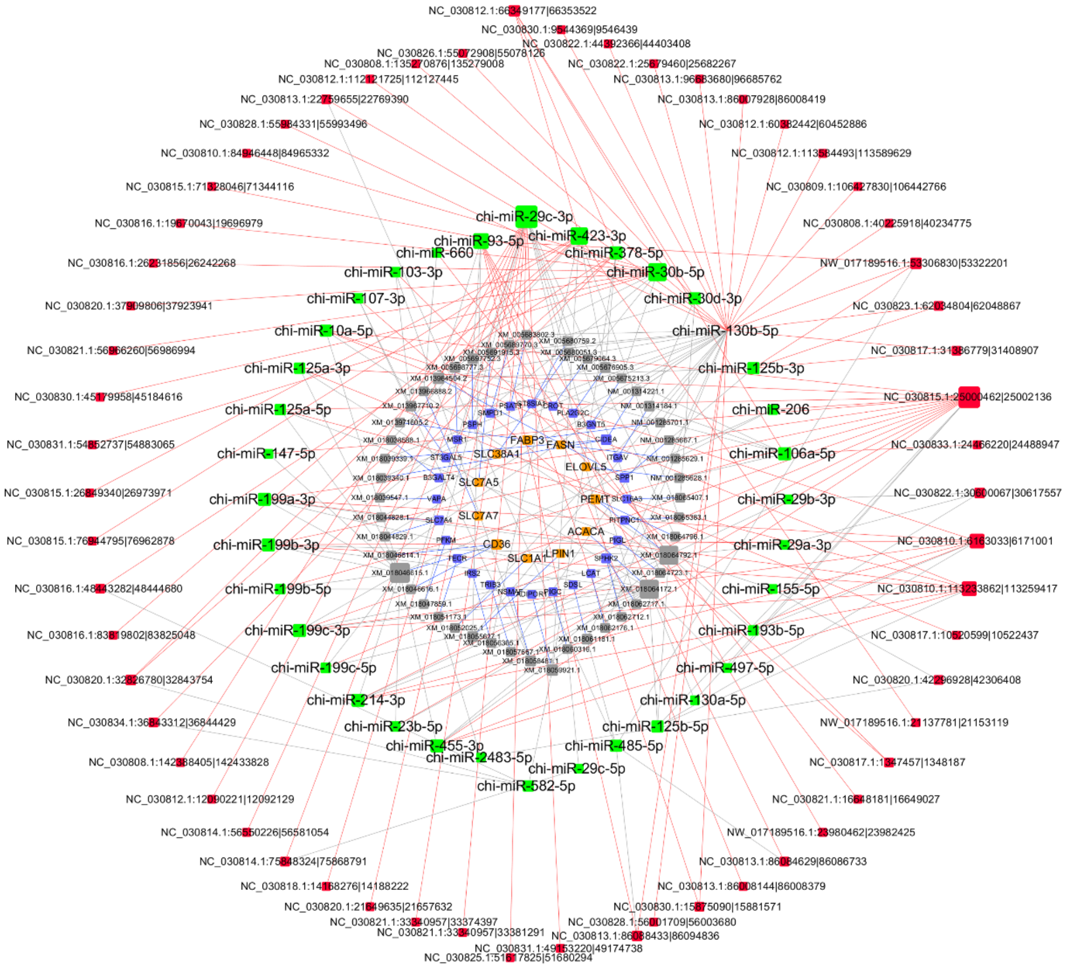

**Figure S14.** Analysis of circRNA-miRNA-mRNA ceRNA regulatory network related to mammary gland substance metabolism.

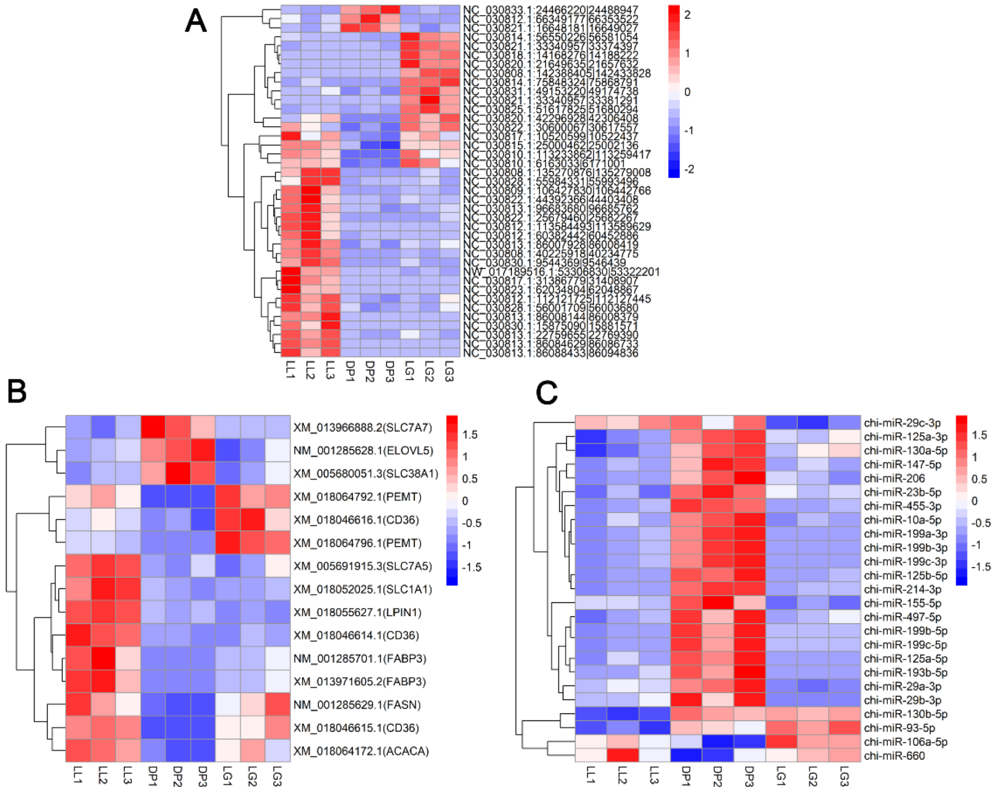


**Figure S15.** Expression heat map of mammary gland substance metabolism-related circRNAs, mRNAs, and miRNAs. (**A**) Expression heat map of circRNAs related to mammary gland substance metabolism; (**B**) Expression heat map of mRNAs related to mammary gland substance metabolism; (**C**) Expression heat map of miRNAs related to mammary gland substance metabolism.

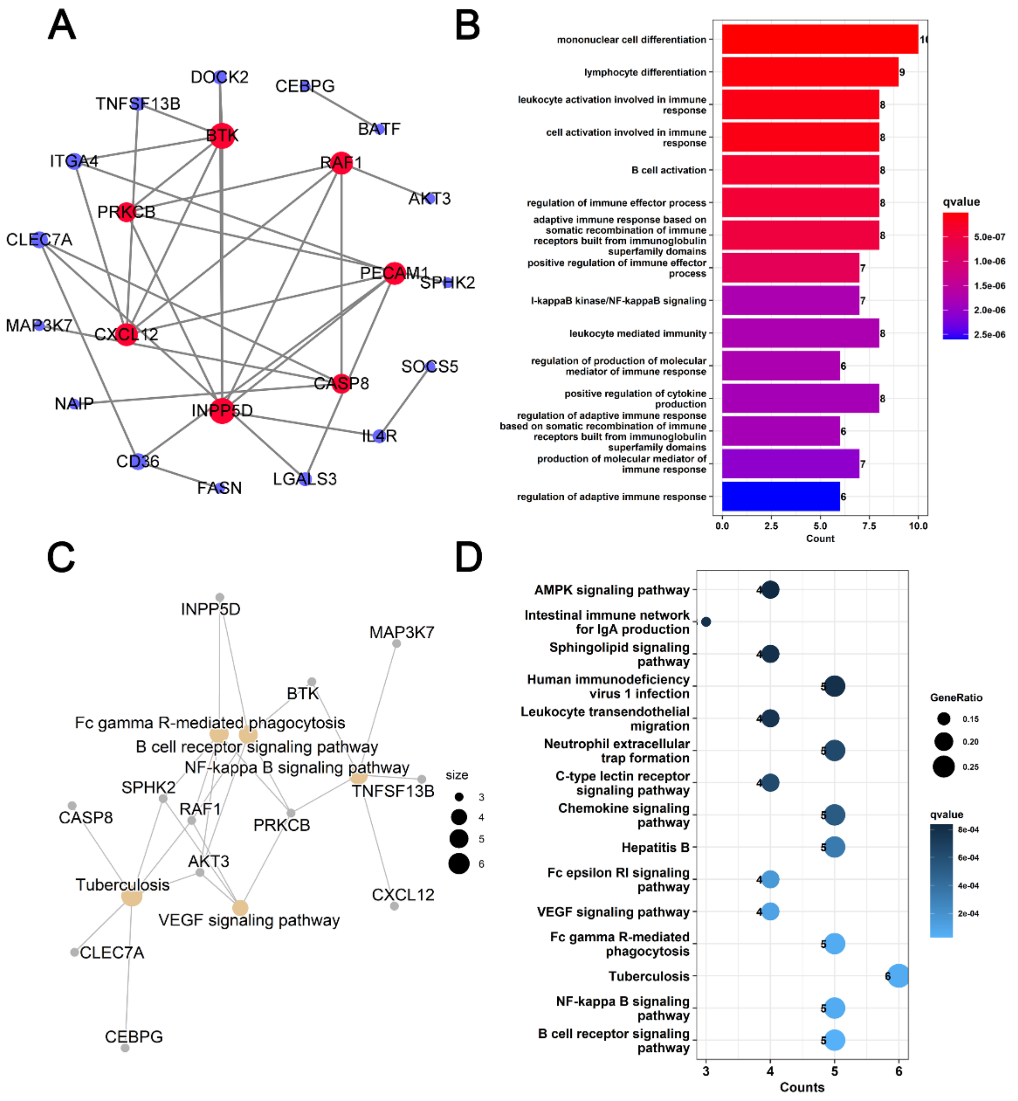


**Figure S16.** Analysis of immune-related circRNA-miRNA-mRNA ceRNA regulatory network in mammary gland. (**A**) PPI network analysis of mammary immune-related genes; (**B**) GO analysis of mammary immune-related genes; (**C**) Relationship between KEGG pathways and genes; (**D**) KEGG analysis of mammary immune-related genes.

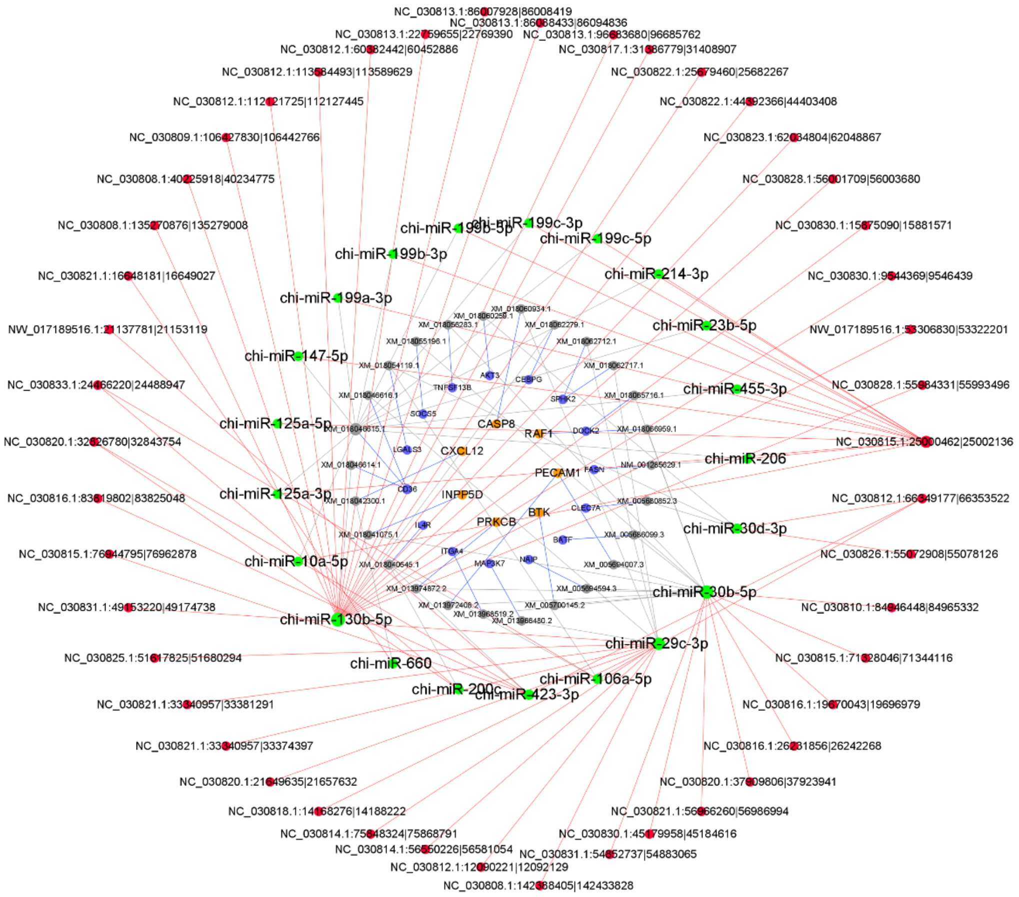


**Figure S17.** Analysis of circRNA-miRNA-mRNA ceRNA regulatory network related to mammary gland immunity.

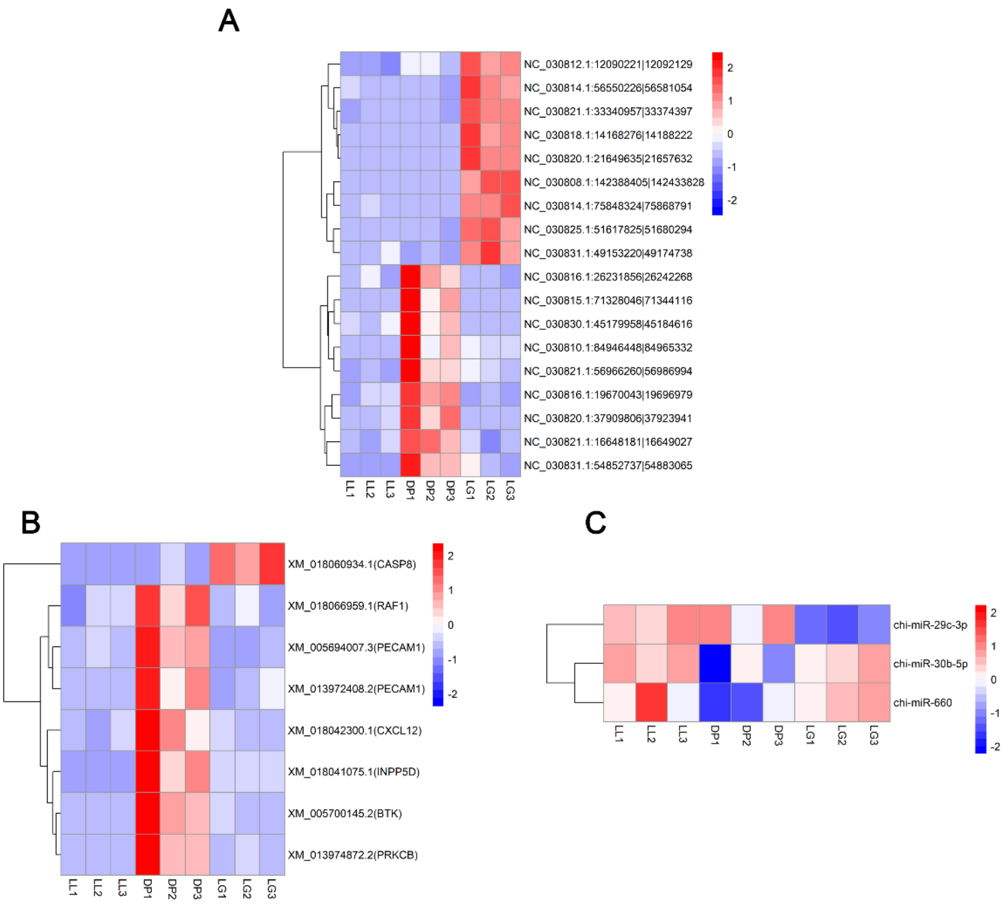


**Figure S18.** Expression heat map of mammary immune-related circRNAs, mRNAs, and miRNAs. (**A**) Expression heat map of mammary immune-related circRNAs; (**B**) Expression heat map of mammary immune-related mRNAs; (**C**) Expression heat map of mammary gland immune-related miRNAs.

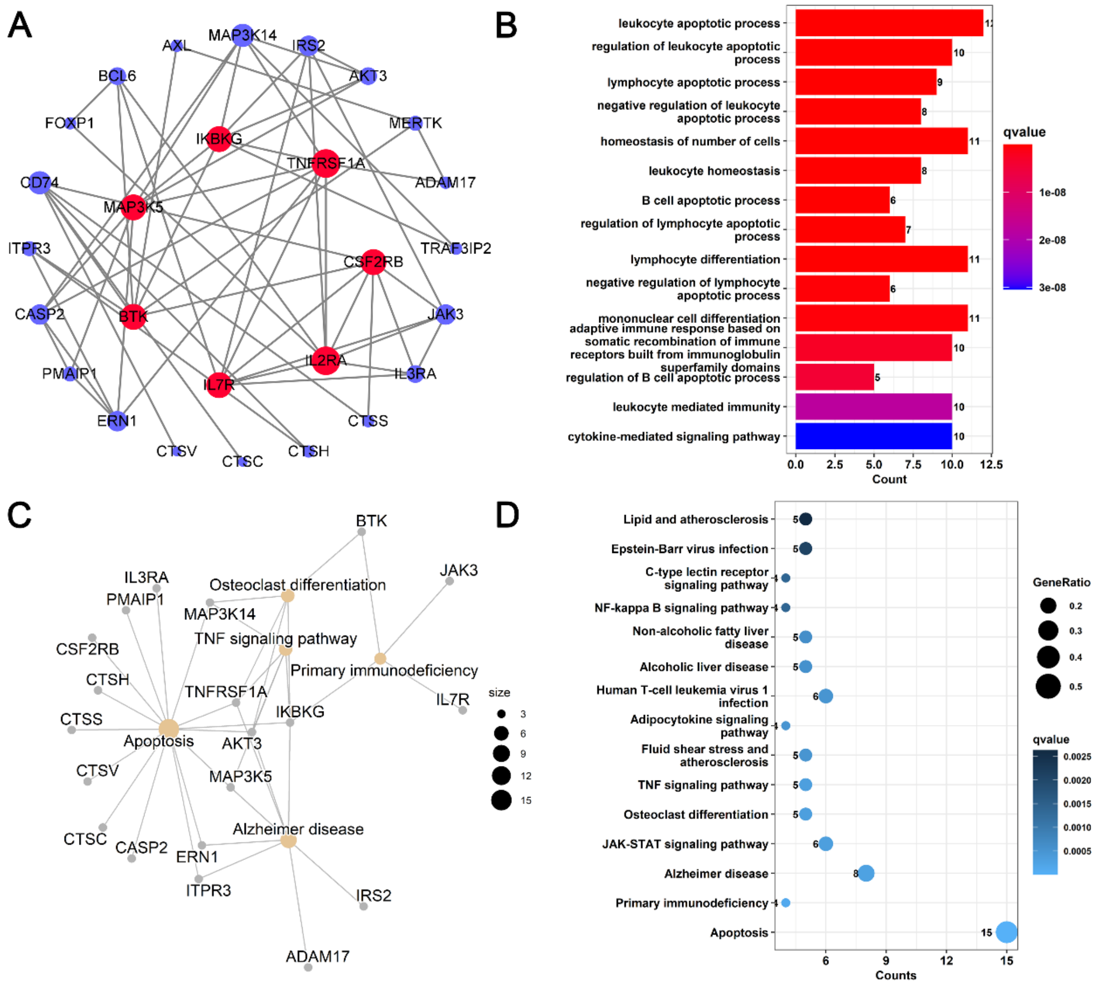


**Figure S19.** Analysis of mammary cell apoptosis-related circRNA-miRNA-mRNA ceRNA regulatory network. (**A**) PPI network analysis of mammary cell apoptosis-related genes; (**B**) GO analysis of mammary cell apoptosis-related genes; (**C**) The relationship between KEGG pathways and genes; (**D**) KEGG analysis of mammary cell apoptosis-related genes.

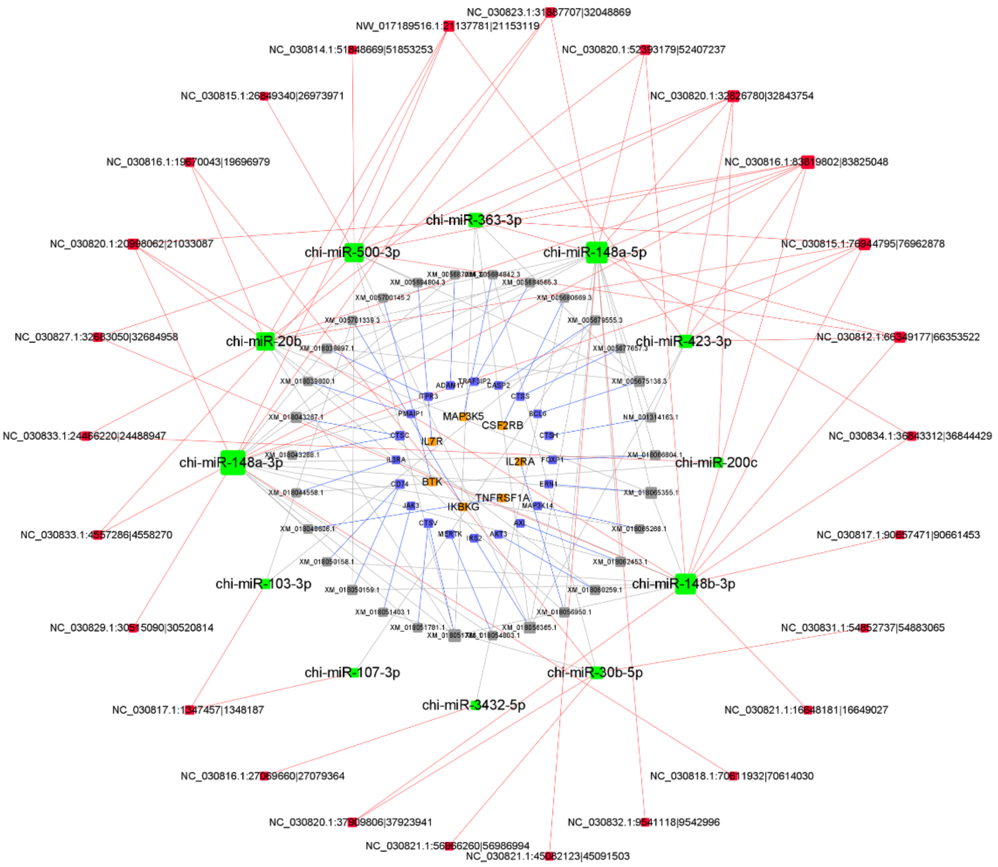


**Figure S20.** Analysis of circRNA-miRNA-mRNA ceRNA regulatory network related to mammary cell apoptosis.

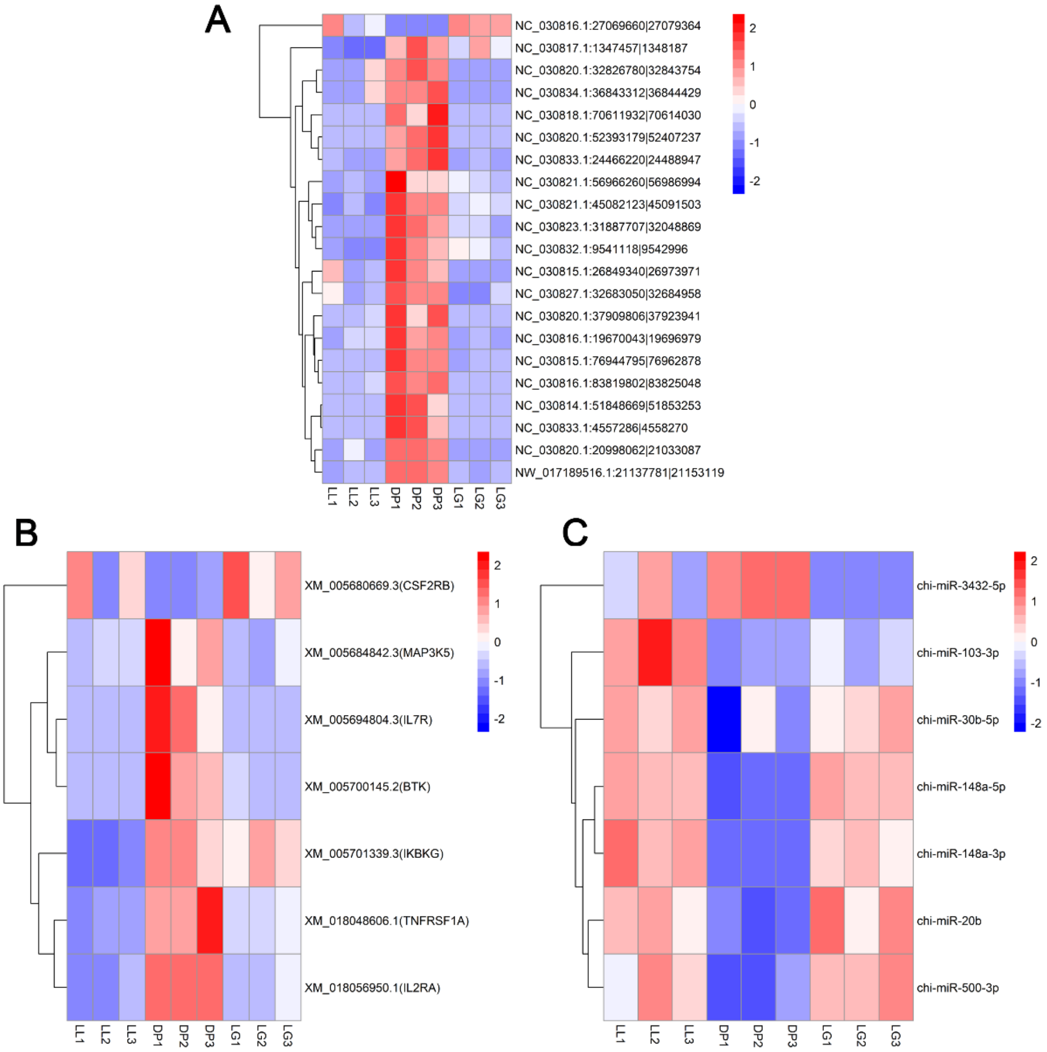


**Figure S21.** Expression heat map of mammary cell apoptosis-related circRNAs, mRNAs and miRNAs. (**A**) Expression heat map of mammary cell apoptosis-related circRNAs; (**B**) Expression heat map of mammary cell apoptosis-related mRNAs; (**C**) Expression heat map of mammary cell apoptosis-related miRNAs.
