# Supplementary material for: Identification and Characterization of circRNAs in Non-Lactating Dairy Goat Mammary Glands Reveal Their Regulatory Role in Mammary Cell Involution and Remodeling
Source: Biomolecules. 2023 May 18;13(5):860. doi: 10.3390/biom13050860 (PMC10216389; doi:10.3390/biom13050860)
Supplement: Supplementary file 1 [file biomolecules-13-00860-s001.zip › Supplementary materials/Supplementary Materials Tables Titles.docx]

**Supplementary Tables Titles**

Table S1. Basic statistics of sequencing results.

Table S2. Source genes and classification of circRNAs.

Table S3. GO functional annotation and KEGG pathway enrichment analysis of circRNA source genes.

Table S4. Results of circRNAs differential expression analysis.

Table S5. Primer sequences of circRNAs.

Table S6. The prediction results of the targeting relationship between miRNAs and circRNAs or miRNAs and mRNAs.

Table S7. Clustering and functional analysis of differentially expressed circRNAs.

Table S8. Analysis of circRNA-miRNA-mRNA ceRNA regulatory network related to mammary gland development.

Table S9. Analysis of circRNA-miRNA-mRNA ceRNA regulatory network related to substance metabolism.

Table S10. Analysis of immune-related circRNA-miRNA-mRNA ceRNA regulatory network in mammary gland.

Table S11. Analysis of mammary cell apoptosis-related circRNA-miRNA-mRNA ceRNA regulatory network.
